# Cholinergic anti-inflammatory pathway ameliorates murine experimental Th2-type colitis by suppressing the migration of plasmacytoid dendritic cells

**DOI:** 10.1038/s41598-021-04154-2

**Published:** 2022-01-07

**Authors:** Yuya Kanauchi, Takeshi Yamamoto, Minako Yoshida, Yue Zhang, Jaemin Lee, Shusaku Hayashi, Makoto Kadowaki

**Affiliations:** Division of Gastrointestinal Pathophysiology, Institute of Natural Medicine, University of Toyama, 2630 Sugitani, Toyama, 930-0194 Japan

**Keywords:** Inflammatory bowel disease, Ulcerative colitis, Mucosal immunology, Enteric nervous system, Plasmacytoid dendritic cells

## Abstract

Ulcerative colitis (UC) is a chronic inflammatory bowel disease. Several studies have demonstrated that α7 nicotinic acetylcholine receptors (α7nAChRs) exert anti-inflammatory effects on immune cells and nicotine suppress UC onset and relapse. Plasmacytoid dendritic cells (pDCs) reportedly accumulate in the colon of UC patients. Therefore, we investigated the pathophysiological roles of α7nAChRs on pDCs in the pathology of UC using oxazolone (OXZ)-induced Th2-type colitis with BALB/c mice. 2-deoxy-D-glucose, a central vagal stimulant suppressed OXZ colitis, and nicotine also ameliorated OXZ colitis with suppressing Th2 cytokines, which was reversed by α7nAChR antagonist methyllycaconitine. Additionally, α7nAChRs were expressed on pDCs, which were located very close to cholinergic nerve fibers in the colon of OXZ mice. Furthermore, nicotine suppressed CCL21-induced bone marrow-derived pDC migration due to Rac 1 inactivation, which was reversed by methyllycaconitine, a JAK2 inhibitor AG490 or caspase-3 inhibitor AZ-10417808. CCL21 was mainly expressed in the isolated lymphoid follicles (ILFs) of the colon during OXZ colitis. The therapeutic effect of cholinergic pathway on OXZ colitis probably through α7nAChRs on pDCs were attributed to the suppression of pDC migration toward the ILFs. Therefore, the activation of α7nAChRs has innovative therapeutic potential for the treatment of UC.

## Introduction

Ulcerative colitis (UC) is a chronic inflammatory disease of the colon. It is commonly assumed that the pathogenesis in UC involves T helper (Th) 2-dominant immune responses^1^. However, the distinct pathogenesis of UC remains to be elucidated.

Over the past several decades, it has been reported that the morbidity of UC in smokers is lower than that in nonsmokers^2,3^ and that most UC patients who have a smoking history are diagnosed with UC after smoking cessation^4^. Moreover, transdermal nicotine attenuates symptoms of UC^5,6^. Therefore, nicotine is considered to suppress the onset and relapse of UC. On the other hand, it is known that many patients with Crohn's disease showing Th1-dominant immune responses have a smoking habit^7^ and that smoking is a major risk factor for recurrence of Crohn’s disease^8^. Accordingly, it is assumed that the effect of nicotine on inflammatory bowel disease (IBD) depends on its immunological background. However, it is difficult to directly use nicotine for the treatment of UC because nicotine often induces adverse drug reactions^5^.

Recently, neuroimmune interactions with important roles in homeostatic mechanisms have been shown to function well in the gut, but the morphological and pathophysiological interactions between immune cells and enteric neurons in the gut still remain obscure. There are several lines of studies on the cholinergic anti-inflammatory pathway activated by the vagus nerve and alpha 7 nicotinic acetylcholine receptors (α7nAChRs) in immune-mediated gastrointestinal diseases^9–14^. Stimulation of the vagus nerve and activation of α7nAChRs on macrophages inhibits the release of inflammatory cytokines from activated macrophages^9,10,15^. It has been reported that 2-deoxy-D-glucose (2-DG), a drug widely used as a central vagal stimulant in some animal species^16^ including mice^17^, significantly suppresses LPS-enhanced permeability in the rat colon, which is blocked by atropine or vagotomy^18^. In addition, we have already reported that vagal stimulation with 2-DG alleviates the allergic symptoms in a murine food allergy model, which is reversed by the nAChR antagonist hexamethonium^11^.

Furthermore, we previously demonstrated that nicotine or α7nAChR agonist suppress the development of dextran sulfate sodium (DSS)-induced colitis, one of the best described and most widely used animal models of UC^19^, which is largely consistent with a report using three α7nAChR agonists in DSS colitis model^20^. Thus, it is suggested that nicotine ameliorates UC via the activation of α7nAChRs on immune cells. However, it has been reported that α7nAChR knock-out mice develop similar degree of DSS colitis as in littermate wild-type mice^21^, even though vagotomized mice develop a more severe DSS colitis compared with control mice treated with DSS^21,22^. Taking together, it is suggested that the involvement of α7nAChRs in the cholinergic anti-inflammatory pathway is still controversial in experimental colitis models and the pathophysiological roles of α7nAChRs in intestinal inflammation remain under investigation.

Although α7nAChR is a homopentameric ligand-gated and cation-selective ion channel mediating fast synaptic transmission in neurons, de Jonge et al. have reported that α7nAChRs also activate alternative signaling pathways in non-neuronal cells, particularly immune cells^9,12^. Owing to the dual ionotropic/metabotropic functions, the activation of α7nAChRs is suspected to exert potent anti-inflammatory effects such as through controlling cytokine production in non-neuronal cells.

It has been reported that JAK2-STAT3 pathway is involved in cholinergic anti-inflammatory effects through α7nAChRs after vagus nerve stimulation in macrophages^9,12^ and liver cells^23^. However, STAT3 negatively regulates inflammatory responses primarily through anti-inflammatory effects of IL-10, an anti-inflammatory cytokine, without directly suppressing transcription of pro-inflammatory cytokines^24^. Thus, it is suggested that the activation of α7nAChRs may interfere with other signaling pathways such as NF-κB and ERK in immune cells^12^.

Therefore, the activation of α7nAChRs is assumed to directly or indirectly trigger the activation of various intracellular signaling pathways, eventually exerting anti-inflammatory effects in immune cells. However, to date, the precise and detailed mechanisms remain to be fully elucidated.

Dendritic cells (DCs) are professional antigen-presenting cells that play pivotal roles in the initiation of acquired immune responses^25^ by migrating to appropriate sites with appropriate timing. After antigen exposure, DCs phagocytose antigens in peripheral tissues and migrate via the afferent lymphatic vessels into the draining lymph nodes to stimulate naïve T cells^25^. The migratory abilities of DCs are of fundamental importance for their functions, but their underlying molecular mechanisms are still largely unknown.

DCs are roughly divided into conventional DCs (cDCs) and plasmacytoid DCs (pDCs)^26^. There are several reports concerning the involvement of pDCs in the pathology of human UC and experimental UC models. The number of pDCs in the colonic mucosa of patients with UC is increased^27^. Moreover, pDCs play a critical role in the development of colitis in animal models^28,29^. However, the pathophysiological roles of pDCs and nicotinic acetylcholine receptors (nAChRs) on pDCs in the pathology of UC remain unclear.

We hypothesized that nicotine attenuates the symptoms of UC through the activation of nAChRs, especially α7nAChRs on pDCs. Therefore, the present study was undertaken to investigate the pathophysiological roles of α7nAChRs on pDCs in the pathology of UC by using a murine oxazolone (OXZ)-induced Th2-dominant colitis model^30^ with BALB/c mice, a Th2-dominant strain, but not C57BL/6, a Th-1 dominant strain, and to elucidate the underlying molecular mechanisms, which leads to an understanding of the pathological mechanisms of UC, thereby facilitating the development of innovative therapeutic agents for UC treatment.

## Results

### Elucidation of OXZ colitis

BALB/c mice intrarectally injected with OXZ exhibited weight loss (supplementary Fig. 1A), congestion in the peritoneal cavity, megacolon, and disruption of the epithelial layer associated with mucosal ulceration. The disease activity score (DAS) and the macroscopic colonic damage score (CDS) of OXZ-induced colitis mice (OXZ mice) were increased (the values in normal mice are zero; DAS: 8.7 ± 1.9, CDS: 8.7 ± 3.4, *n* = 3), as with our previous report^31^. In the present study, the mortality rate of OXZ colitis mice was 28.7 ± 6.0% on day 4 (70 mice from seven independent experiments).

The mRNA expression of the Th1 cytokine IFN-γ was significantly decreased, whereas that of the Th2 cytokines IL-4, IL-5 and IL-10 was significantly increased in the spleen of OXZ mice (supplementary Fig. 1B). On the other hand, the mRNA expression of all Th1 and Th2 cytokines was significantly increased in the middle colon of OXZ mice (supplementary Fig. 1C).

BALB/c mice, a Th2-dominant strain, showed weight loss, while C57BL/6 mice, a Th1-dominant strain, failed to show weight loss following OXZ treatment (supplementary Fig. 2A). The DAS and CDS of BALB/c mice (DAS: 9.3 ± 1.3, CDS: 6.8 ± 0.9, supplementary Fig. 2B) were significantly higher than those of C57BL/6 mice (DAS: 2.3 ± 1.9, CDS: 1.4 ± 1.4, supplementary Fig. 2B). The administration of FK506, a calcineurin inhibitor that shifts the Th1/Th2 immune balance toward Th2-dominant immunity^32^, significantly aggravated the disease severity of OXZ colitis (supplementary Fig. 2C,D). These data indicate that Th2-dominant immune responses are the main responses involved in the pathological mechanisms of OXZ colitis.

In UC practice guidelines, patients with mild to moderate UC take oral 5-aminosalicylic acid (5-ASA), and patients with severe UC take oral steroids (e.g., prednisolone). Therefore, we assessed the effects of these drugs on the development of OXZ colitis. 5-ASA failed to suppress OXZ colitis (supplementary Fig. 2E), whereas prednisolone significantly decreased the DAS, the CDS and myeloperoxidase (MPO) activity (DAS: 2.4 ± 1.2, CDS: 2.3 ± 0.5, MPO activity: 0.9 ± 0.3 kunits/gwwt) in OXZ mice compared with vehicle-treated OXZ mice (DAS: 6.9 ± 2.1, CDS: 5.2 ± 1.4, MPO activity: 3.2 ± 0.4 kunits/gwwt) (supplementary Fig. 2F). Therefore, OXZ colitis can mimic moderate or severe UC.

### Effects of vagus nerve stimulation and α7nAChR activation on OXZ colitis

**Figure 1:**
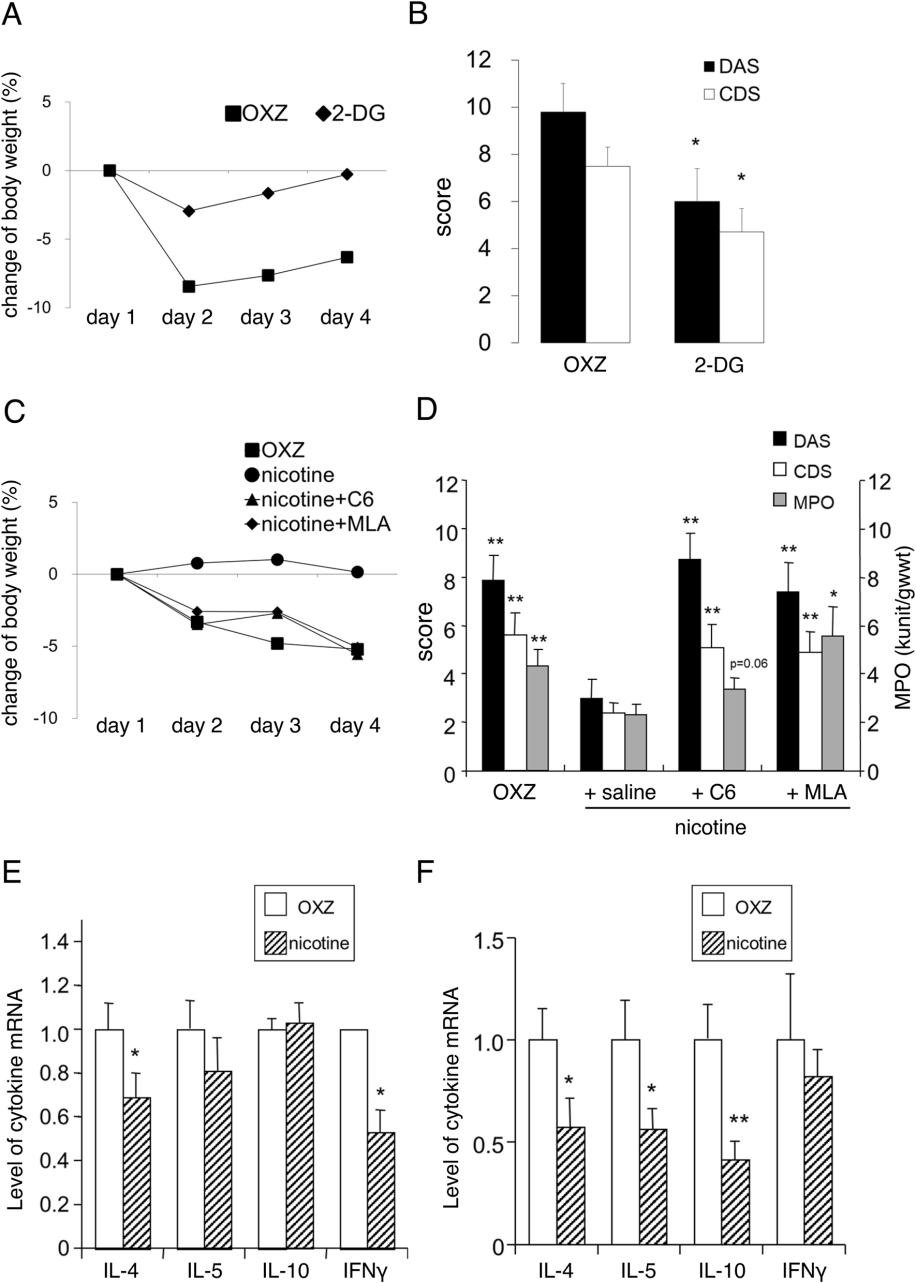
The effect of 2-DG or nicotine on OXZ mice was evaluated. (**A**) Stimulation of the vagus nerve by 2-DG inhibited weight loss in OXZ mice (The typical mean weight change during the development of colitis is shown). 2-DG at a dose of 200 mg/kg was intraperitoneally administered 1 h before the intrarectal injection of the OXZ solution on day 1 and given one time daily on day 2 and 3 at the same time as day 1. (**B**) The DAS and CDS in OXZ mice treated with 2-DG (*n* = 19) were decreased compared with those in OXZ mice (*n* = 27) (*P < 0.05 vs OXZ mice). (**C**, **D**) The effect of nAChR activation by nicotine (1 mg/kg) on OXZ colitis and the effect of pretreatment with C6 or MLA before the administration of nicotine to OXZ mice was evaluated. Nicotine (1 mg/kg) was subcutaneously administered 1 h before the intrarectal injection of the OXZ solution on day 1 and given one time daily on day 2 and 3 at the same time as day 1. C6 (32 mg/kg) and MLA (1 mg/kg) were subcutaneously administered 30 min before the administration of nicotine at the same time on day 1, 2 and 3. Nicotine inhibited weight loss in OXZ mice and C6 or MLA reversed the suppressive effects of nicotine on weight loss (**C**; the typical mean weight change during the development of colitis is shown). The DAS, the CDS, and MPO activity in OXZ mice treated with nicotine were decreased compared with those in OXZ mice, which were reversed by C6 or MLA (**D**; DAS: *n* = 15–16, CDS: *n* = 15–16, MPO activity: *n* = 7–14, *P < 0.05, ** *P* < 0.01 vs nicotine-treated OXZ mice). (**E**, **F**) The mRNA expression of IL-4, IL-5, IL-10, and IFN-γ in the spleen (**E**) and middle colon (**F**) of OXZ mice with or without nicotine treatment was measured. Nicotine significantly decreased the mRNA expression of IL-4 and IFN-γ in the spleen (**E**, *n* = 8–21, *P < 0.05 vs OXZ mice) and that of IL-4, IL-5, and IL-10 in the middle colon (**F**, *n* = 4–9, *P < 0.05, **P < 0.01 vs OXZ mice). Data are represented as the mean value ± SEM. *P* values were calculated using 2-tailed unpaired Student’s *t*-test (**B**, **E**, **F**) or one-way ANOVA with Dunnett’s multiple comparison test (**D**).

We investigated the effect of the cholinergic anti-inflammatory pathway on the development of OXZ colitis. Since the entire colon of rodents is reportedly innervated by the vagus nerve from the proximal to the distal portion^33^, we undertook an experiment to stimulate the vagus nerve in the OXZ colitis model. Stimulation of the vagus nerve by administration of 2-DG reduced weight loss (Fig. 1A) and significantly decreased the DAS and the CDS (DAS: 6.0 ± 1.4, CDS: 4.7 ± 1.0) in OXZ mice compared with vehicle-treated OXZ mice (DAS: 9.8 ± 1.2, CDS: 7.5 ± 0.8) (Fig. 1B). Next, we examined the effect of nAChR activation on OXZ colitis. The activation of nAChRs by the administration of nicotine dose-dependently suppressed the development of OXZ colitis at doses of 0.32–3.2 mg/kg. Pretreatment with hexamethonium (C6), a nonspecific nAChR antagonist, or methyllycaconitine (MLA), an α7nAChR antagonist, significantly reversed the suppressive effects of nicotine on weight loss, the DAS (nicotine: 3.0 ± 0.8, C6: 8.7 ± 1.1, MLA: 7.4 ± 1.2), the CDS (nicotine: 2.4 ± 0.4, C6: 5.1 ± 1.0, MLA: 4.9 ± 0.9), and MPO activity (nicotine: 2.3 ± 0.5, C6: 3.4 ± 0.5, MLA: 5.5 ± 1.2 kunits/gwwt) in OXZ colitis (Fig. 1C,D). However, MLA failed to suppress OXZ colitis by itself (DAS: OXZ colitis 6.8 ± 1.2 (*n* = 9) vs MLA 6.8 ± 1.3 (*n* = 10); CDS: OXZ colitis 5.0 ± 1.0 (*n* = 9) vs MLA 5.3 ± 0.9 (*n* = 10)). The mRNA expression of IL-4 and IFN-γ in the spleen was significantly decreased in nicotine-treated OXZ mice compared with vehicle-treated OXZ mice (Fig. 1E). On the other hand, the mRNA expression of IL-4, IL-5, and IL-10 in the middle colon was significantly decreased in nicotine-treated OXZ mice compared with vehicle-treated OXZ mice (Fig. 1F).

These data indicate that activation of the cholinergic anti-inflammatory pathway via the vagus nerve exerts suppressive effects on OXZ colitis, which is in good agreement with the previous report that electrical stimulation of the vagus nerve improves a mortality rate in an acute and severe type of OXZ colitis model^14^. Furthermore, nicotine ameliorates OXZ colitis through α7nAChRs, which is mainly attributed to the suppression of enhanced Th2-dominant mucosal immunity in the colon of OXZ mice.

### Localization and identification of α7nAChR-expressing cells in the colon

**Figure 2:**
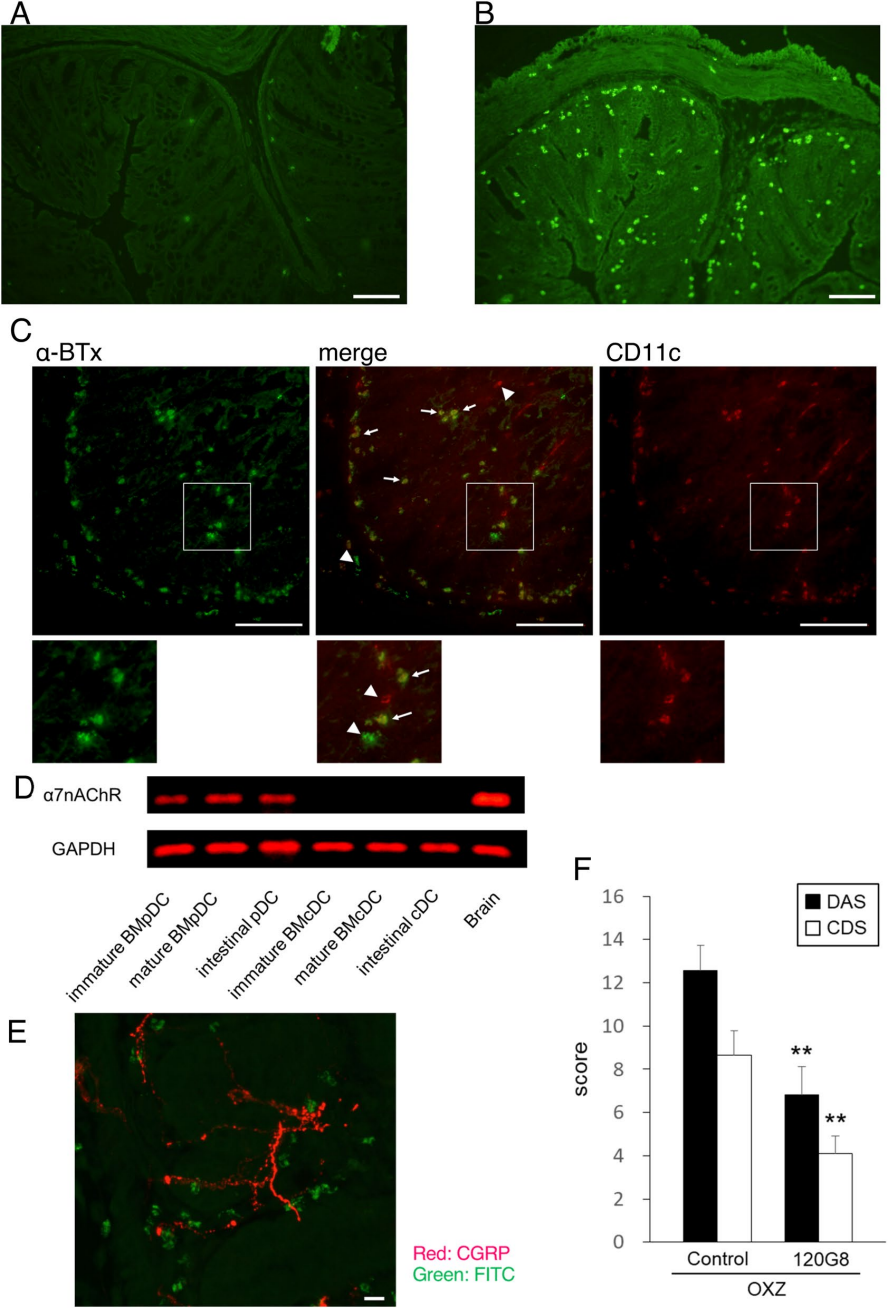
The localization of α7nAChRs in the lamina propria was determined by immunohistochemistry. (**A**) FITC-α-BTx-binding cells were sparse in the middle colon of normal mice (a representative result is shown). (**B**) The number of FITC-α-BTx-binding cells was dramatically elevated in the middle colon of OXZ mice (a representative result is shown). (**C**) A large number of FITC-α-BTx-binding cells expressed CD11c (arrows in images of both lower and higher magnification). Arrowheads indicate FITC-α-BTx single binding cells or CD11c single positive cells. A representative result is shown. (**D**) The mRNA expression of α7nAChRs in DCs was measured by RT-PCR (a representative result is shown). The full-length gel is shown in supplementary Fig. 3. mPDCA-1^+^CD11C^int^ pDCs collected from the lamina propria of the middle colon in OXZ mice, and immature and mature mouse BMpDCs expressed α7nAChR mRNA. (**E**) A large number of FITC-α-BTx-binding cells were located in close vicinity of the CGRP-immunoreactive cholinergic enteric nerve fibers (a representative result is shown). The scale bars represent 100 μm in (**A**–**C**) and 10 μm in (**E**). (**F**) pDC depletion with the anti-120G8 antibody significantly ameliorated OXZ colitis (*n* = 9–10, **P < 0.01 vs OXZ mice). Data are represented as the mean value ± SEM. *P* values were calculated using 2-tailed unpaired Student’s *t*-test.

We examined the localization of α7nAChRs in the lamina propria of the colon using a FITC-conjugated α-bungarotoxin (FITC-αBTx), which has the ability to selectively bind to α7nAChRs. FITC-α-BTx-binding cells were sparse in the middle colon of normal mice (Fig. 2A), while the number of FITC-α-BTx-binding cells was dramatically elevated in the middle colon of OXZ mice (Fig. 2B). Furthermore, although FITC-α-BTx single binding cells or CD11c single positive cells were observed (Fig. 2C; arrowheads in images of both lower and higher magnification), a large number of FITC-α-BTx-binding cells also expressed CD11c (Fig. 2C; arrows in images of both lower and higher magnification), suggesting that α7nAChRs are expressed on DCs in the colon of OXZ mice.

Next, we examined the mRNA expression of α7nAChRs in DCs by semiquantitative RT-PCR. All mPDCA-1^+^CD11C^int^ pDCs collected from the colonic lamina propria of OXZ mice, immature bone marrow-derived pDCs (BMpDCs) and mature BMpDCs expressed α7nAChR mRNA (Fig. 2D; the full-length gel is shown in supplementary Fig. 3). However, little expression of α7nAChR mRNA was observed in cDCs collected from the colonic lamina propria of OXZ mice, immature BMcDCs or mature BMcDCs.

Next, we investigated the morphological interaction between pDCs and cholinergic enteric nerve fibers in the middle colon of OXZ mice. It has been reported that cholinergic neurons comprise 70–80% of all enteric neurons in the mouse^34^, and that in the mouse enteric nervous system, calcitonin gene-related peptide (CGRP) is exclusively contained in intrinsic primary afferent neurons that are immunoreactive for choline acetyltransferase^34^, indicating that CGRP-immunoreactive neurons contain ACh as a neurotransmitter in the mouse colon. FITC-α-BTx-binding cells were found in the colonic lamina propria of OXZ mice, many of which were located in close vicinity of the CGRP-immunoreactive cholinergic enteric nerve fibers (Fig. 2E).

Furthermore, the pDC-depleting antibody 120G8^35^ was used to investigate the pathogenetic role of pDCs in the development of OXZ colitis. pDC depletion with the anti-120G8 antibody significantly ameliorated OXZ colitis (OXZ mice: DAS 12.6 ± 1.2, CDS 8.7 ± 1.1, *n* = 9; anti-120G8 antibody-treated OXZ mice: DAS 6.8 ± 1.3, CDS 4.1 ± 0.8, *n* = 10; *P* < 0.01, Fig. 2F).

Taken together, these results indicate that pDCs play a pathogenetic role in the development of OXZ colitis and that nicotine and vagus nerve stimulation may attenuate colitis through the activation of α7nAChRs expressed on pDCs in the colon.

### Effects of α7nAChR activation on pDC immunogenic functions

In general, after peripheral antigen uptake, mature DCs migrate as specialized antigen-presenting cells from the peripheral tissues to the draining lymph nodes via the afferent lymphatic vessels to induce the activation and differentiation of naïve T cells. Chemokine-activating signaling via chemokine receptors expressed by mature DCs guide DCs toward the draining lymph nodes where chemokines are mainly expressed.

Firstly, the activation of nAChRs failed to affect the maturation and uptake capability of pDCs (supplementary Fig. 4).

**Figure 3:**
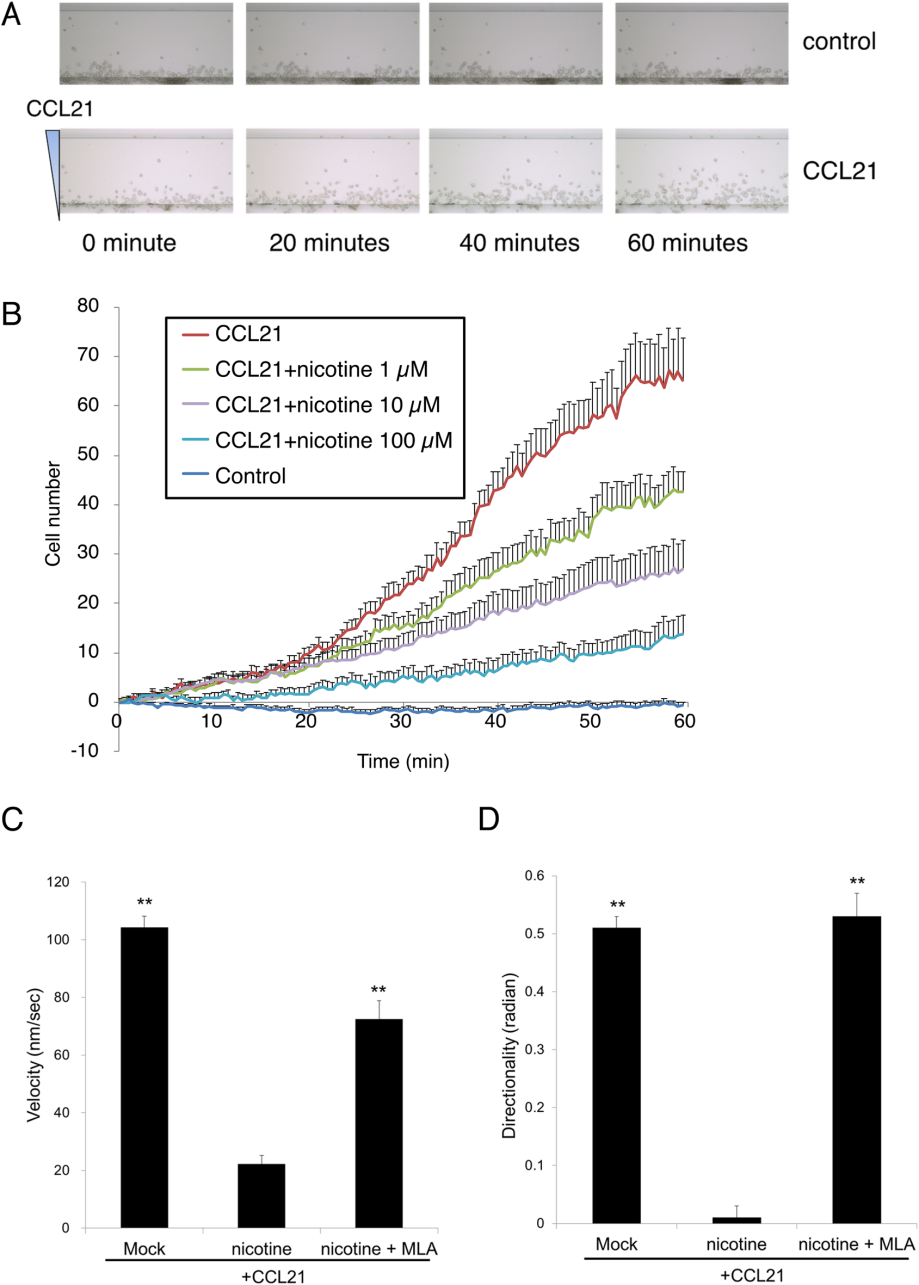
The inhibitory effect of nicotine on BMpDC migration was evaluated with a chemotaxis assay. (**A**) BMpDCs matured by treatment with CpG oligodeoxynucleotides chronologically migrated in response to an established concentration gradient of CCL21 (a representative experiment is shown). (**B**) The number of migrating BMpDCs was dose-dependently inhibited by the addition of nicotine (1–100 μM) to the chemotaxis assay medium (*n* = 3–7). (**C**, **D**) The velocity and directionality of BMpDC migrating in response to CCL21 were calculated. The velocity and directionality of BMpDC migration were significantly inhibited by the addition of nicotine (10 µM) to the chemotaxis assay medium, and pretreatment with MLA blocked the inhibitory effect of nicotine (*n* = 20 for each group, **P < 0.01 vs nicotine). Data are represented as the mean value ± SEM. *P* values were calculated using one-way ANOVA with Dunnett’s multiple comparison test (**C**, **D**).

Thus, we examined the effect of nicotine on CCL21-induced BMpDC migration. We performed a chemotaxis assay with BMpDCs matured by treatment with CpG oligodeoxynucleotides. BMpDCs migrated in response to an established concentration gradient of CCL21 (Fig. 3A), and the number of migrated BMpDCs was dose-dependently reduced by nicotine at doses of 1–100 μM (Fig. 3B). In addition, the velocity and directionality of migrating BMpDCs were calculated. The velocity and directionality of BMpDC migrating in response to CCL21 were 104.2 ± 4.0 nm/s and 0.51 ± 0.02 radians, respectively, which were significantly inhibited by nicotine in the chemotaxis assay medium (final concentration 10 μM, velocity: 22.1 ± 3.2 nm/s, directionality: 0.01 ± 0.02 radians, Fig. 3C,D). Moreover, MLA significantly reversed the inhibitory effect of nicotine (velocity: 72.5 ± 6.4 nm/s, directionality: 0.53 ± 0.04 radians, Fig. 3C,D).

**Figure 4:**
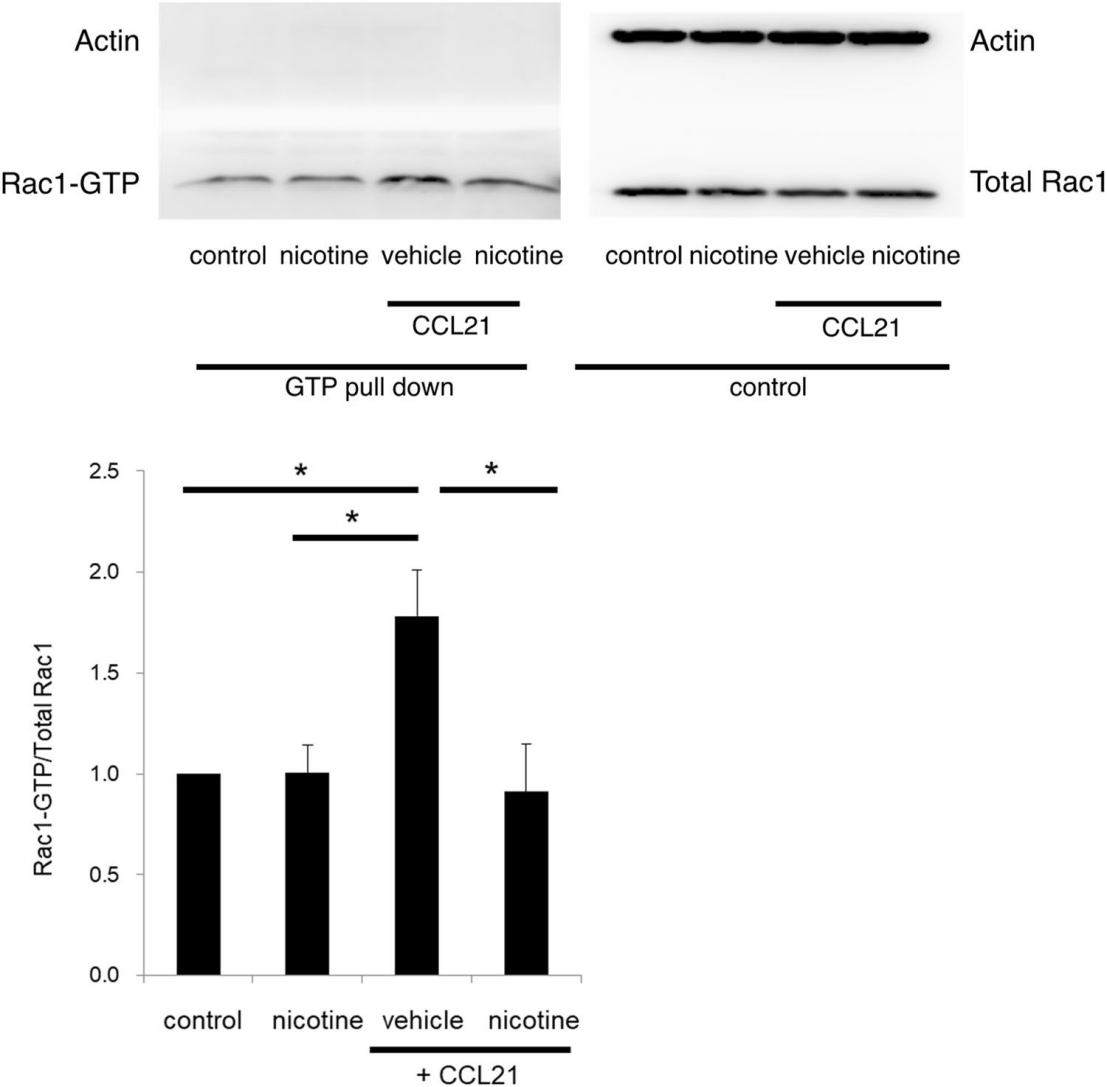
The expression of active Rac 1 (Rac1-GTP) and total Rac 1 in BMpDCs was measured by western blotting. The ratio of active Rac 1 to total Rac 1 was significantly increased by stimulation with CCL21, and the increase in the ratio induced by CCL21 was inhibited by pretreatment with nicotine (10 µM) (a representative band pattern is shown; *n* = 4 for each group, *P < 0.05 vs CCL21). The full-length blots are shown in supplementary Fig. 5. Data are represented as the mean value ± SEM. *P* values were calculated using one-way ANOVA with Dunnett’s multiple comparison test.

Furthermore, activation of Rac 1, a Rho GTPase family member, is essential for DC migration^36^. We examined the expression of active and total Rac 1 in BMpDCs by western blotting. The ratio of active Rac 1 (Rac1-GTP) to total Rac 1 was significantly increased by stimulation with CCL21, but this increase was prevented by pretreatment with nicotine (Fig. 4; the full-length blots are shown in supplementary Fig. 5).

These data demonstrated that α7nAChR activation by nicotine suppressed BMpDC migration due to the inactivation of Rac1.

**Figure 5:**
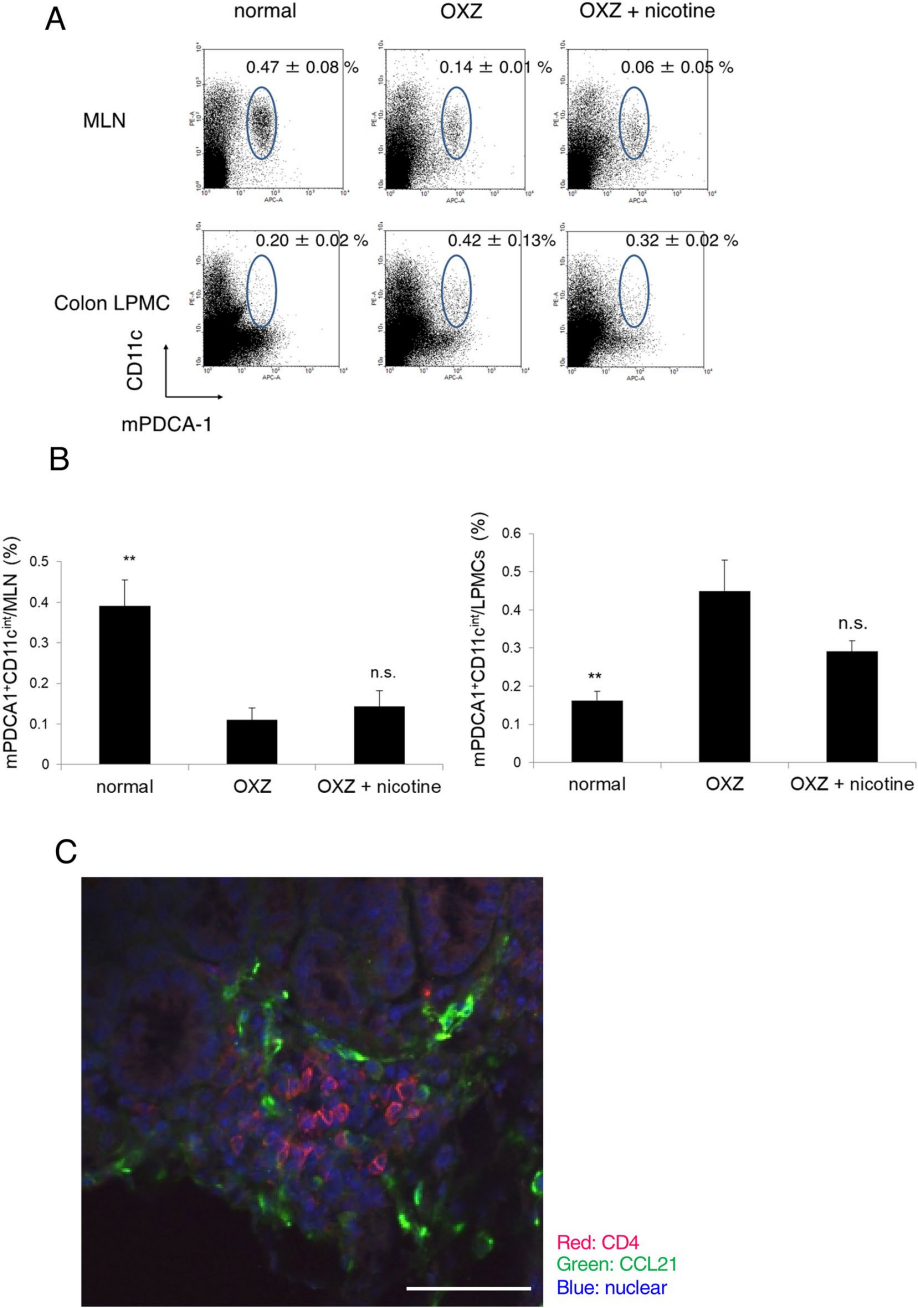
The proportions of pDCs in the colonic mucosa and MLN of OXZ mice were analyzed. (**A**, **B**) The proportions of pDCs in the MLN and LPMCs of the middle colon in normal mice, OXZ mice, and OXZ mice treated with nicotine were compared. The frequency of pDCs in the MLN of OXZ mice was significantly decreased compared with that in the MLN of normal mice. The frequency of pDCs in LPMCs from OXZ mice was increased compared with that in the LPMCs from normal mice. The frequencies of pDCs in the MLN and LPMCs of OXZ mice treated with nicotine were equivalent to the corresponding frequencies of OXZ mice (**A**: a representative experiment is shown, **B**: *n* = 4–6, **P < 0.01 vs OXZ, n.s.: not significant). (**C**) The localization of CCL21 in OXZ mice was investigated by immunohistochemistry. CCL21 was mainly expressed around the T cell zone in ILFs (a representative result is shown). The scale bars represent 50 μm. Data are represented as the mean value ± SEM. *P* values were calculated using one-way ANOVA with Dunnett’s multiple comparison test (**B**).

Consequently, we investigated the accumulation of pDCs in the mesenteric lymph node (MLN) and colonic mucosa. The proportion of pDCs in the colonic mucosa of OXZ mice was significantly increased compared with that in the colonic mucosa of normal mice (Fig. 5A,B), indicating enhanced recruitment of pDCs to inflamed sites. Furthermore, the proportion of pDCs in the MLN of OXZ mice was markedly decreased compared with that in the MLN of normal mice. These findings are in close agreement with the previous finding that DSS treatment drives pDC accumulation in the colonic mucosa but conversely decreases the number of pDCs in the MLN^29^. In contrast, neither the pDC proportion in the MLN nor that in the colonic mucosa was significantly affected by nicotine treatment during OXZ colitis, regardless of the severity of the colitis symptoms (Fig. 5A,B), suggesting the trafficking of mature pDCs within the inflamed colon but not to the MLN.

Next, we investigated the localization of CCL21 in sites within the colon other than the MLN. CCL21 was mainly expressed around the T cell zone in the isolated lymphoid follicles (ILFs) of the middle colon during OXZ colitis (Fig. 5C). Taken together, these results indicate that mature pathogenic pDCs mainly migrate to ILFs but not the MLN for antigen presentation to naïve T cells and drive the subsequent differentiation of these T cells into Th2 cells in the ILFs, thereby causing OXZ colitis.

### Mechanisms underlying the inhibition of pDC migration by nicotine

**Figure 6:**
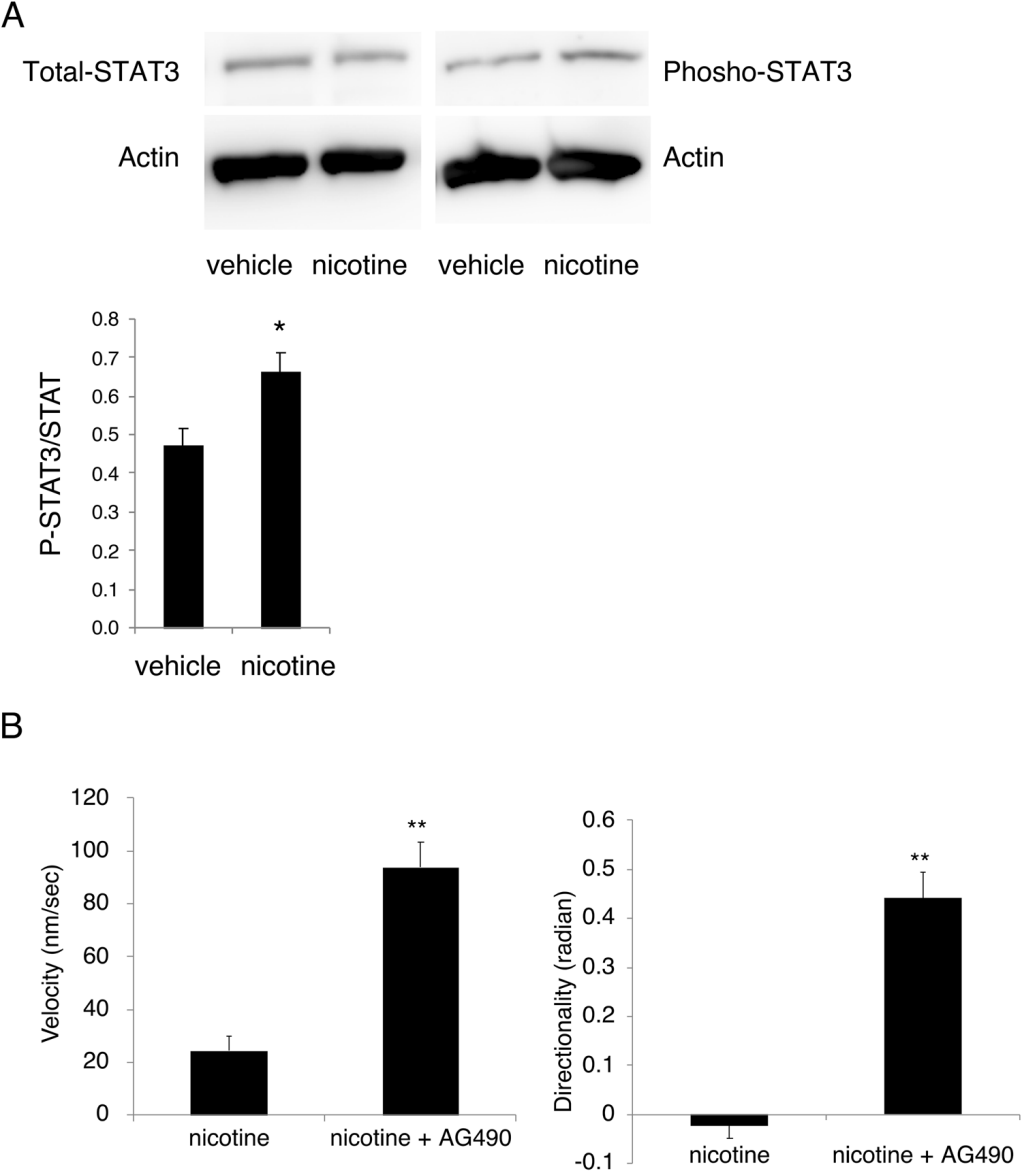
The involvement of the JAK2-STAT3 signaling pathway in the inhibitory effect of nicotine on BMpDC migration was examined. (**A**) The ratio of phosphorylated STAT3 to total STAT3 in BMpDCs was significantly increased by stimulation with nicotine (10 µM), as determined by immunoblotting (a representative band pattern is shown; *n* = 3 for each group, *P < 0.05 vs vehicle). The full-length blots are shown in supplementary Fig. 6. (**B**) Pretreatment with AG490, a JAK2 inhibitor, blocked the inhibitory effect of nicotine (10 µM) on BMpDC migration velocity and directionality in a chemotaxis assay (*n* = 10 for each group, **P < 0.01 vs nicotine). Data are represented as the mean value ± SEM. *P* values were calculated using 2-tailed unpaired Student’s *t*-test one-way (**A**, **B**).

We investigated the involvement of JAK2-STAT3 signaling pathway in the suppressive effect of nicotine on pDC migration by immunoblotting because α7nAChR activation reportedly activates the JAK2-STAT3 pathway^9,12^. The ratio of phosphorylated STAT3 to total STAT3 in BMpDCs was significantly elevated by activation with nicotine (Fig. 6A; the full-length blots are shown in supplementary Fig. 6). In addition, pretreatment with AG490, a JAK2 inhibitor, significantly reversed the suppressive effects of nicotine on BMpDC migration velocity (nicotine: 24.4 ± 5.4 nm/s, nicotine + AG490: 93.9 ± 9.2 nm/s) and directionality (nicotine: − 0.02 ± 0.03 radians, nicotine + AG490: 0.44 ± 0.05 radians) in the chemotaxis assay (Fig. 6B), suggesting that the activation of the JAK2-STAT3 signaling pathway is involved in the mode of action for the inhibitory effect of nicotine.

Furthermore, it has been reported that the expression and proteolytic activity of caspase-3 are enhanced by STAT3 activation in the muscles of tumor-bearing mice^37^ and that caspase-3 may play versatile nonlethal functions in many cell types^38^.

**Figure 7:**
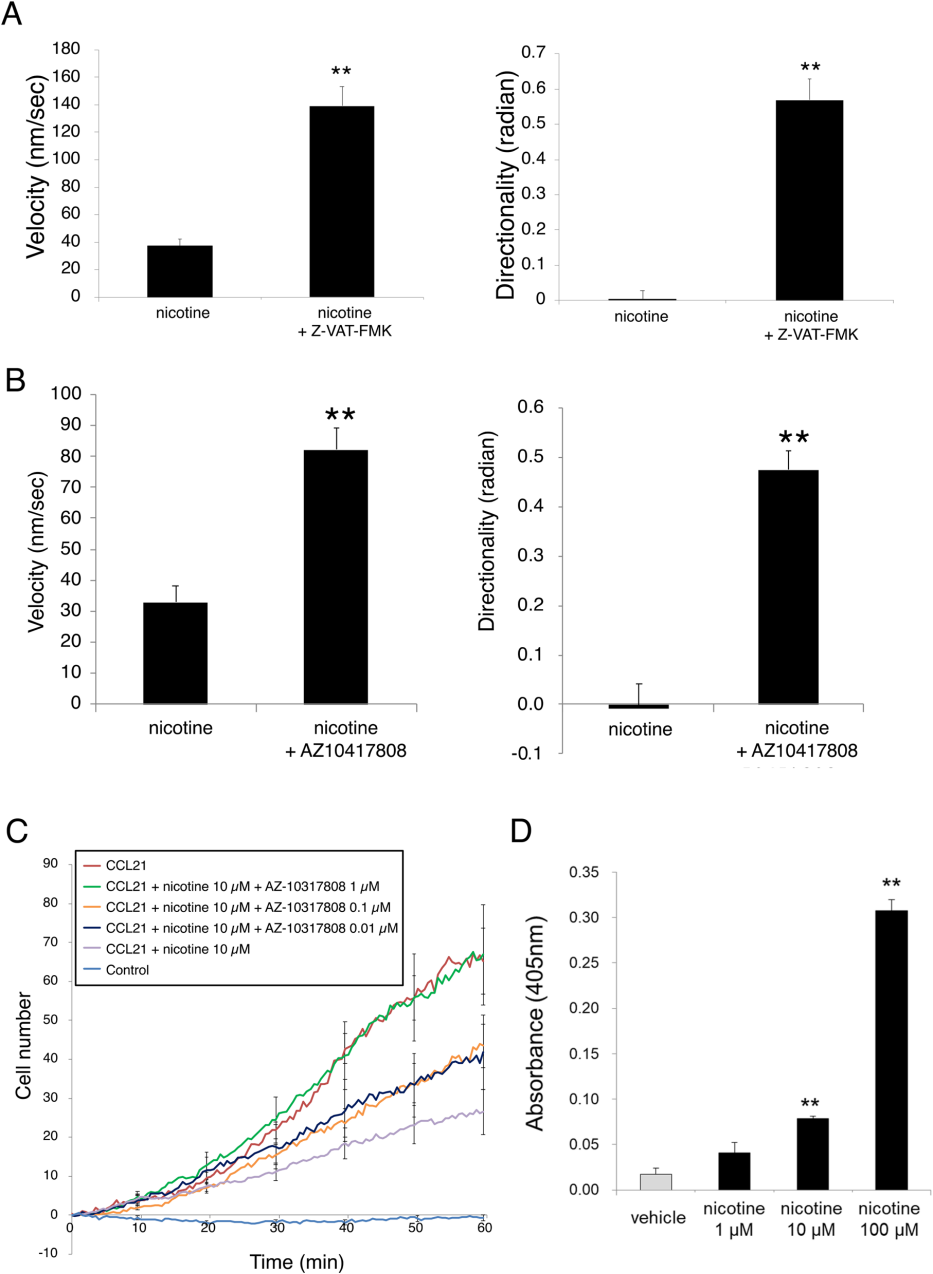
The involvement of caspase-3 in the effect of nicotine on BMpDC migration was evaluated. (**A**) Pretreatment with Z-VAD-FMK, a nonselective caspase inhibitor, blocked the inhibitory effect of nicotine (10 µM) on BMpDC migration velocity and directionality (*n* = 10 for each group, **P < 0.01 vs nicotine). (**B**) Pretreatment with AZ-10417808, a selective caspase-3 inhibitor, blocked the inhibitory effect of nicotine (10 µM) on BMpDC migration velocity and directionality (*n* = 10 for each group, **P < 0.01 vs nicotine). (**C**) AZ-10417808 (0.01–1 μM) dose-dependently blocked the inhibitory effect of nicotine on the number of migrated BMpDCs (*n* = 3–7). (**D**) Nicotine (1–100 μM) dose-dependently upregulated caspase-3 activity in BMpDCs in an enzyme-linked immunosorbent assay (*n* = 3 for each group, **P < 0.01 vs vehicle). Data are represented as the mean value ± SEM. *P* values were calculated using 2-tailed unpaired Student’s *t*-test (**A**, **B**) or one-way ANOVA with Dunnett’s multiple comparison test (**D**).

Therefore, we investigated the involvement of caspases in the suppressive effect of nicotine on pDC migration. Pretreatment with Z-VAD-FMK, a nonselective caspase inhibitor, significantly reversed the suppressive effects of nicotine on BMpDC migration velocity (nicotine: 37.3 ± 5.0 nm/s, nicotine + Z-VAD-FMK: 139.1 ± 14.0 nm/s) and directionality (nicotine: 0.01 ± 0.01 radians, nicotine + Z-VAD-FMK: 0.51 ± 0.04 radians) (Fig. 7A). Moreover, AZ-10417808, a selective caspase-3 inhibitor, significantly inhibited the effects of nicotine on BMpDC migration velocity (nicotine: 32.9 ± 5.4 nm/s, nicotine + AZ-10417808: 82.2 ± 7.0 nm/s) and directionality (nicotine: − 0.01 ± 0.05 radians, nicotine + AZ-10417808: 0.47 ± 0.04 radians) (Fig. 7B). Furthermore, AZ-10417808 dose-dependently suppressed the effect of nicotine (0.01–1 μM) on the number of migrated BMpDCs (Fig. 7C).

In addition, nicotine treatment did not induce apoptosis in BMpDCs (Supplementary Fig. 7) but dose-dependently upregulated the nonapoptotic activity of caspase-3 in BMpDCs at doses of 1–100 μM in an enzyme-linked immunosorbent assay (Fig. 7D).

## Discussion

The pathology of UC is primarily attributed to excessive Th2 immune responses, even if Th1 immune responses are involved in the pathogenesis of UC to some extent. OXZ colitis is considered to closely resemble UC in some aspects, such as clinical features, histological features and in particular immunological features that are shifted toward Th2-dominant immunity in the colon^39^. Thus, we employed OXZ colitis to elucidate the therapeutic effects of the cholinergic anti-inflammatory pathway mediated through α7nAChRs on a Th2-dominant colitis model mimicking UC.

Although mammalian immunity is composed of the mucosal and systemic immune systems, the mucosal immune system is thought to be the system primarily involved in the pathology of UC.

In the present study, immune responses were shifted toward Th2 dominance in the systemic immune system, and Th2 immune responses were significantly enhanced in the mucosal immunity mainly involved in OXZ colitis. The pathology of OXZ colitis mediated primarily by Th2 immune responses was verified by the employment of C57BL/6 mice and treatment with FK506 in OXZ colitis.

Our results revealed that nicotine significantly reduced the enhanced expression of IL-4 but not that of other Th2 cytokines in the spleen of OXZ mice and significantly suppressed the expression of every Th2 cytokine but not that of Th1 cytokine in the colon of OXZ mice, suggesting that the administration of nicotine ameliorates OXZ colitis mainly by suppressing the enhanced Th2 immune responses involved in mucosal immunity.

The cross-talk between the autonomic nervous system and immune system, especially the mucosal immune system, is increasingly being elucidated to be of great importance for the maintenance of immune homeostasis in the gut. To date, several reports indicate that the vagus nerve plays a counterregulatory role in the digestive system to restore immune homeostasis in various inflammatory disease models, such as DSS-induced colitis^21,22^, postoperative ileus^10^, pancreatitis^40^, and food allergy^11^. Furthermore, it is thought that the anti-inflammatory effects of the vagus nerve on these murine experimental models are mostly attributed to the suppression of proinflammatory cytokine release by immune cells. Recently, α7nAChRs, which are expressed on mouse DCs^41^, macrophages^9,10,12,15^, T cells^19^ and mucosal mast cells^11^, and human mononuclear cells^42^, have been regarded as a main molecule in the cholinergic anti-inflammatory pathway via the vagus nerve^43^. However, little is known about whether and how cholinergic activation though α7nAChRs on immune cells in the colon serve as anti-inflammatory pathways in gut Th2-related inflammatory diseases.

In the present study, vagal stimulation with 2-DG or activation of α7nAChRs by nicotine ameliorated OXZ colitis. However, MLA by itself had no effect on OXZ colitis, suggesting that the cholinergic pathway through α7nAChRs may not continuously exert anti-inflammatory effects on Th2-type OXZ colitis, but only when α7nAChRs are activated. Furthermore, to our knowledge, for the first time, the present results revealed that α7nAChRs were expressed on pDCs but not cDCs, although other nAChRs are reportedly expressed on cDCs^44^. Our morphological findings of the close proximity between α7nAChR-expressing pDCs and cholinergic enteric nerve fibers in the colon of OXZ mice imply that the neural cholinergic regulation of immune responses is mediated via α7nAChRs on immune cells, which is consistent with previous reports^13^, including our report on DSS colitis model^19^ and food allergy model^11^.

Recently, the pathophysiological role of pDCs in colonic inflammation has gradually been elucidated. Notably, pDCs are reported to accumulate in the inflamed colon of mice with DSS colitis^29^ and patients with UC^27^.

To date, it remains unclear whether and how pDCs are involved in the pathogenic mechanisms of IBD. In the present study, pDC depletion significantly increased the survival rate of OXZ mice, which is in close agreement with the suppressive effect of selectively ablating pDCs on the development of DSS colitis^29^. However, pDCs stimulated by a gut commensal molecule show anti-inflammatory effects on 2,4,6-trinitrobenzene sulfonic acid (TNBS)-induced Th1-type colitis by driving IL-10 production by CD4^+^ T cells^45^. Thus, it is suggested that the pathophysiological role of pDCs depends on the immunological background of each colitis model.

Nicotine upregulates the expression of MHC class II, CD40, CD54, CD86 and LFA-1 on human cDCs derived from peripheral monocytes with GM-CSF^44^; however, the effects of nAChR activation on pDC functions have never been reported. In the present study, the activation of nAChRs on pDCs did not affect the expression of CD80 or CD86 or antigen uptake (supplementary Fig. 4B,D), suggesting that pDCs differ from cDCs in regard to the effects of nicotine on maturation and antigen uptake.

Recent studies have underlined the importance of DC migration in the maintenance of immune defense mechanisms and immune homeostasis and in the pathogenic mechanisms of immune diseases^25^. Immature DCs sample and process self and nonself antigens and thereafter undergo activation. Subsequently, the interactions between CC-chemokine receptors on mature DCs and CC-chemokine ligands enable mature DCs to eventually migrate into the draining lymph node, especially the T cell-rich paracortex. It is assumed that migratory mature pDCs in the intestine traffic to the MLN in a manner dependent on the interaction of CCL19/21 and CCR7^46,47^. Although inhibition of lymphocyte migration into the colon is an effective treatment for UC^48^, it remains unclear whether pDC migration is involved in the pathogenesis of UC.

The present study demonstrated that the activation of α7nAChRs inhibited the migration of BMpDCs, suggesting that the inhibition of pDC migration through the activation of α7nAChRs suppresses the development of OXZ colitis.

Taken together, these results indicate that pDC migration is essential for the pathogenic mechanism of OXZ colitis.

Furthermore, in the present study, the accumulation of pDCs was markedly decreased in the MLN of OXZ mice and increased in the colonic mucosa of OXZ mice. Interestingly, to the best of our knowledge, we demonstrated that ILFs express CCL21 in the colon of OXZ mice, which is in good agreement with a previous experimental finding in the colon of DSS colitis mice^49^ and a previous clinical finding in humans that CCL21 is rarely expressed in lymph follicles of the normal colons, but is detected in lymph follicles of the inflamed colons in patients with IBD^50^. Furthermore, we and Cruickshank et al. have reported that CD11c^+^B220^+^ pDCs are located in the colonic ILFs of colitis mice^49,51^. In addition, Cruickshank et al. have demonstrated that some CD11c^+^ cells are in close contact with CD4^+^ T cells in the colonic ILFs of colitis mice^51^. Taken all together, these findings suggest that mature pathogenic pDCs mainly migrate into the ILFs but not the MLN for antigen presentation to naïve T cells and subsequently drive the subsequent differentiation of Th2 CD4^+^ T cells in the ILFs, thereby causing OXZ colitis. Therefore, the activation of α7nAChRs inhibits the migration of pathogenic pDCs into ILFs in the colonic mucosa of OXZ mice, thereby suppressing the activation of Th2 CD4^+^ T cells in ILFs, their homing to inflamed sites in the colonic mucosa of OXZ mice and, eventually, the development of colitis.

The migration of DCs is controlled by chemokines and their receptors^52^. The interaction between CCR7 expressed on DCs and CCL19/21 produced by the lymphatic system is required for DC migration into the draining lymph nodes^46,47^, and the Rho family, including Rho, Rac and Cdc42, is involved in the activation of the CCR7-CCL19/21 signaling pathway^53^. Rac1, although not required for DC differentiation and maturation, plays indispensable roles in the biogenesis of dendrites and migration of mature DCs^36^. In addition, Rac 1 and dedicator of cytokinesis protein (DOCK) 2 play essential roles, especially in the chemotaxis of pDCs^54^. DOCK can operate as a regulator of pDC migration in the upstream of Rac 1 by controlling the activation of Rac 1^55^. Moreover, the numbers of pDCs in the spleen, peripheral lymph node and MLN but not those of cDCs are decreased in DOCK2-deficient mice^54^. Accordingly, DOCK2-Rac 1 signaling is indispensable for the migration of pDCs but not that of cDCs.

The present study shows the suppressive effect of nicotine on Rac 1 activation, indicating that the suppression of pDC migration by nicotine can be attributed to its suppression of DOCK2-Rac 1 signaling.

Pharmacological studies were undertaken to elucidate the signaling downstream of α7nAChR activation to the inactivation of Rac 1. α7nAChR signaling acts as an anti-inflammatory pathway in macrophages^9,10,12,15^. Nicotine and vagus nerve stimulation can activate α7nAChRs on macrophages, thereby inhibiting the transcriptional activity of NF-κB through the activation of the JAK2-STAT3 pathway and eventually suppressing the release of TNF-α from macrophages^9,12^. However, it has never been reported that JAK-STAT signaling through α7nAChRs is closely involved in pDC migration.

The present study demonstrated that STAT3 phosphorylation in BMpDCs was upregulated by nicotine treatment and that the inhibition of JAK2 by AG490 dramatically suppressed the inhibitory effect of nicotine on pDC migration, indicating that α7nAChR activation induces an enhancement in JAK2-STAT3 signaling not only in macrophages but also in pDCs.

Furthermore, it has been reported that STAT3 binds to the caspase-3 promoter, thereby increasing the expression of pro-caspase-3^37^. Caspase family members, especially caspase-3, are mainly involved in apoptosis. However, it is noteworthy that caspases do not necessarily cause cell death and can serve as indispensable enzymes for a wide range of nonlethal cellular functions in a variety of cellular processes, such as signaling, proliferation, differentiation, remodeling and neuronal plasticity^38^.

Rac1 can be cleaved and inactivated by caspase 3 in lymphocytes^56^ and COS-7 cells^57^, indicating that Rac 1 is also a substrate of caspase-3. Furthermore, caspases reportedly function in Rac-dependent cell motility^58^.

In the present study, we demonstrated that a selective caspase-3 inhibitor abolished the inhibitory effect of nicotine on BMpDC migration. Moreover, nicotine treatment upregulated caspase-3 activity in BMpDCs. Therefore, we conclude that nicotine eventually suppresses Rac1 activation through nonlethal caspase-3 activation, thereby inhibiting pDC migration.

**Figure 8:**
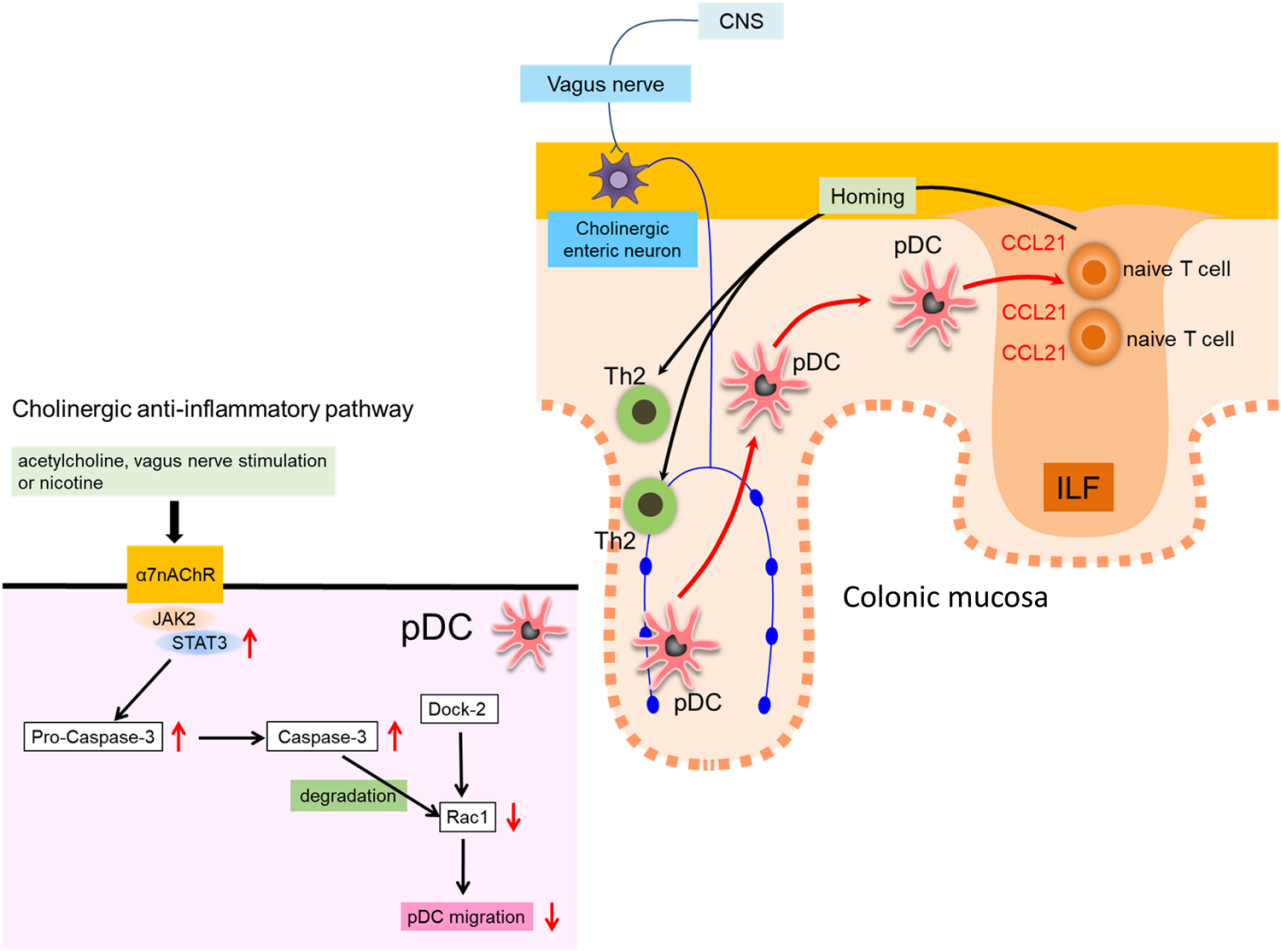
Graphical abstract. The cholinergic anti-inflammatory pathway (nicotine and vagus nerve stimulation) ameliorated Th2-type OXZ colitis through α7nAChRs on pDCs, which was attributed to the suppression of pDC migration toward CCL21 in ILFs of the colonic mucosa of OXZ mice by α7nAChR-mediated JAK2-STAT3 activation, subsequent nonapoptotic caspase-3 activation and eventual Rac 1 inactivation.

In summary (Fig. 8), we demonstrate that nicotine and vagus nerve stimulation suppress the development of Th2-type OXZ colitis probably through the activation of α7nAChRs expressed on pDCs, which is primarily attributed to the suppression of pDC migration into ILFs in the colonic mucosa via the activation of JAK2-STAT3 signaling, subsequent activation of nonapoptotic caspase-3 and eventual inactivation of Rac 1.

To conclude, this study improves the understanding of the pathophysiological roles of the cholinergic anti-inflammatory vagal pathway and α7nAChR signaling in the pathological mechanisms of UC. Furthermore, if these underlying mechanisms are involved in the pathogenesis of human UC, they might represent innovative therapeutic targets for the treatment of human UC.

## Materials and methods

### Chemicals and reagents

FK506 was obtained from Fujisawa Pharmaceutical (Osaka, Japan). Reagents for PCR were purchased from Takara Bio (Shiga, Japan), Nacalai Tesque (Kyoto, Japan), or QIAGEN (Hilden, Germany). CCL21, a goat IgG anti-mouse CCL21 antibody, AZ-10417808, GM-CSF and FLT3 ligand were purchased from R&D Systems (Minneapolis, MN, USA). Antibodies were purchased from Miltenyi Biotec (Bergisch Gladbach, Germany), BioLegend (San Diego, CA, USA), Covance (Princeton, NJ, USA), or BD Biosciences (San Jose, CA, USA). CpG oligodeoxynucleotide 2216 was purchased from Hokkaido System Science (Sapporo, Japan). An Annexin V PI apoptosis detection kit was purchased from Beckman Coulter (Brea, CA, USA). The Active Rac 1 Pull-Down and Detection Kit was purchased from Life Technologies (Carlsbad, CA, USA). AG-490 was purchased from Merck KGaA (Darmstadt, Germany). The Caspase-3/CPP32 Colorimetric Assay Kit was purchased from BioVision (Milpitas, CA, USA). The 120G8 antibody was purchased from DENDRITICS (Dardilly, France). Others were purchased from Sigma-Aldrich (St. Louis, MO, USA) or FUJIFILM Wako (Osaka, Japan).

### Animals

Four-week-old male BALB/c and C57BL/6 mice were purchased from Japan SLC (Shizuoka, Japan). In the present study, BALB/c mice were used for all experiments except for the comparison of susceptibility between BALB/c mice and C57BL/6 mice in the OXZ colitis model. All mice were housed in the experimental animal facility at the University of Toyama. This study was carried out in compliance with the ARRIVE guidelines and approved by the Animal Experiment Committee of the University of Toyama (Authorization No. A2018NM-3 and A2015NM-2) in accordance with the Guide for the Care and Use of Laboratory Animals at the University of Toyama, which is accredited by the Ministry of Education, Culture, Sports, Science and Technology, Japan. Following the guidelines, mice were humanely sacrificed by cervical dislocation.

### Induction of colitis

Colitis was induced according to a previously described method^31,59^. Briefly, the abdomen of mice was shaved, and shortly thereafter, a 3% (w/v) OXZ solution was swabbed onto the shaved area. Seven days later (day 1), a 0.5% OXZ solution was intrarectally injected into the mice using a polyurethane catheter. To more accurately assess the severity of the colitis, we improved the previously reported assessment method^31,59^. Using this assessment method, the colitis was assessed on day 4 by assessing DAS, CDS and MPO activity. Body weight and disease symptoms were assessed on 3 subsequent days (day 2–4) after the colitis was induced. The mice were sacrificed on day 4, and their tissues were excised and used for morphological and biological analyses. Symptom assessment of the colitis was according to the criteria for the disease activity score (DAS). The DAS is represented by the sum of highest score of the followed each criterion in 3 days (day 2–4): Weight loss (1: 1–5%, 2: 5–10%, 3: 10–20%, 4: more than 20%); Fecal consistency (2: loose feces, 3: mild diarrhea, 4: severe diarrhea); Rectal bleeding (3: mild bleeding, 4: severe bleeding). When animals died, the DAS is scored as 15. At necropsy, colonic damage was assessed according to the criteria for the macroscopic scoring of colonic damage (CDS) based on the degree of inflammation and the presence of edema and/or ulcerations. The CDS is represented by the sum of highest score of the followed each criterion: Tissue damage (1: hyperemia, 2: bleeding or erosion, 3: one site of ulceration or severe erosion, 4: severe ulceration or tissue necrosis); Adhesion (1: mild adhesion, 2: adhesions involving several bowel loops); Mega-intestine (1: narrowness, 2: mega-intestine) on day 4. When animals died, the CDS is scored as 11. The entire colon and spleen were harvested from OXZ mice on day 4. The middle colons next to the ulcer sites in the distal colons of OXZ colitis mice were used for gene expression analysis and morphological analysis and flow cytometry analysis, and the entire colons were used for the measurement of MPO activity.

Test substances were administered to OXZ mice before and after intrarectal injection of the OXZ solution, and the efficacies of these test substances against colitis were evaluated.

Nicotine at doses of 0.32–3.2 mg/kg was subcutaneously administered to the mice once a day. Nicotine was given 1 h before the intrarectal injection of the OXZ solution on day 1 and given on day 2 and 3 at the same time as day 1. 2-DG at a dose of 200 mg/kg was intraperitoneally administered 1 h before the intrarectal injection of the OXZ solution on day 1 and given one time daily on day 2 and 3 at the same time as day 1. Hexamethonium at a dose of 32 mg/kg and MLA at a dose of 1 mg/kg were subcutaneously administered 30 min before the administration of nicotine at the same time on day 1, 2 and 3. FK506, prednisolone, and 5-ASA at doses of 32 mg/kg, 10 mg/kg, and 100 mg/kg, respectively, were orally administered 1 h before the intrarectal injection of the OXZ solution on day 1 and given one time daily on day 2 and 3 at the same time as day 1.

### Depletion of pDCs in the colitis model

pDCs were depleted from OXZ mice by administration of the 120G8 antibody^35^. The 120G8 antibody was intraperitoneally administered at a dose of 150 µg on days 0, 1, and 2. The effect of pDC depletion on developing colitis was assessed on day 4 by determining the DAS, CDS, and survival rate.

### Measurement of mRNA expression

The entire colon and spleen were harvested from OXZ mice on day 4. Total RNA was isolated from the middle colon and spleen by using Sepasol RNA I Super according to the manufacturer’s manuals. Total RNA was subjected to reverse transcription using a PrimeScript RT reagent kit. Amplification of IL-4, IL-5, IL-10, IFN-γ, and GAPDH using cDNA as a template was performed by SYBR Premix Ex Taq. The following primers were used.

IL-4: forward: 5′-GGTCTCAACCCCCAGCTAGT-3′
  reverse: 5′-GCCGATGATCTCTCTCAAGTGAT-3′
IL-5: forward: 5′-GAAGTGTGGCGAGGAGAGAC-3′
  reverse: 5′-GCACAGTTTTGTGGGGTTTT-3′
IL-10: forward: 5′-AGAAGCATGGCCCAGAAATCA-3′
  reverse: 5′-GGCCTTGTAGACACCTTGGT-3′
IFN-γ: forward: 5′-ATGAACGCTACACACTGCATC-3′
  reverse: 5′-CCATCCTTTTGCCAGTTCCTC-3′
GAPDH: forward: 5′-TGACCACAGTCCATGCCATC-3′
  reverse: 5′-GACGGACACATTGGGGGTAG-3′

The expression of target mRNA was normalized to the expression of GAPDH mRNA as an internal control.

### Expression of α7nAChRs

The mRNA expression of α7nAChRs was measured as described below. Total RNA was isolated from BMDCs and colonic DCs by using RNeasy Plus Mini according to the manufacturer’s manuals. Total RNA was subjected to reverse transcription. Amplification of Chrna7 and GAPDH using cDNA as a template was performed. The following primers were used.

GAPDH: forward: 5′-TGACCACAGTCCATGCCATC-3′
  reverse: 5′-GACGGACACATTGGGGGTAG-3′
Chrna7: forward: 5′-AACCATGCGCCGTAGGACA-3′
  reverse: 5′-CTCAGCCACAAGCAGCATGAA-3′

The reaction products were electrophoresed on a 3% agarose gel containing ethidium bromide.

The expression and distribution of α7nAChRs in the colon were investigated according to the following protocol. The middle colons were harvested from OXZ mice and normal mice on day 4 and frozen in cooled n-hexane. Thin frozen sections (7 μm) were cut by using a cryostat microtome (Leica Microsystems, Nussloch, Germany). The sections were washed with ethanol and incubated for 15 min in a FITC-αBTx dissolved in PBS at 4 °C. The sections were fixed with a 4% (w/v) paraformaldehyde (PFA) solution for 15 min at room temperature (RT) and washed with PBS.

### Immunohistochemistry

The colonic sections were treated with a rat IgG anti-mouse CD11c antibody, rat IgG anti-mouse CD4 antibody, rabbit IgG anti-rat CGRP antibody, or goat IgG anti-mouse CCL21 antibody for 12–18 h at 4 °C. After the incubation, the sections were incubated with a suitable secondary antibody for 2 h at RT. The sections were observed using a confocal laser scanning microscope (LSM700; Carl Zeiss Japan, Tokyo, Japan) or a fluorescence microscope (IX71 system; Olympus, Tokyo, Japan) with a U-MWIG3 filter set (Olympus) and photographed using an Olympus digital camera (DP70; Olympus).

### Isolation of lamina propria mononuclear cells

The entire colon was harvested from dissected OXZ mice on day 4. Lamina propria mononuclear cells (LPMCs) were obtained from the entire colon according to a previously described method^60^. Briefly, the colonic epithelium was removed by incubation with 0.5 mM EDTA buffer for 20 min at 37 °C. LPMCs were isolated with an enzymatic dissociation procedure using collagenase. Discontinuous Percoll density-gradient centrifugation was performed to purify LPMCs.

### pDCs and cDCs separation

Colonic pDCs were obtained from LPMCs by using BD IMagnet according to the manufacturer’s manuals. Briefly, a cell suspension of LPMCs was incubated with PE-conjugated anti-mPDCA-1 antibody for 30 min at 4 °C. Subsequently, the suspension was incubated with BD IMag Anti-R-PE Magnetic Particles for 30 min at 4 °C. The colonic pDCs collected from the magnetic reagent-positive fraction were isolated by using BD IMagnet.

Similarly, colonic cDCs were obtained from LPMCs by using BD IMagnet according to the manufacturer’s manuals. A cell suspension of LPMCs was incubated with the Biotinylated Mouse Dendritic Cell EnRichment Cocktail for 15 min at 4 °C. Subsequently, the suspension was incubated with BD IMag Streptavidin Particles Plus for 30 min at 4 °C. From the magnetic reagent-negative fraction, colonic cDCs were isolated by using BD IMagnet. The isolated cells were quantified and purified by sorting with a FACS Aria (BD Biosciences) in certain experiments.

### Differentiation of BMDCs

BMDCs were induced according to a previously reported method^61,62^. BM cells were aseptically obtained from the tibia and femur. BM cells were incubated with 10 ng/mL GM-CSF for 6 d to obtain immature BMcDCs. Similarly, BM cells were incubated with 100 ng/mL FLT3 ligand for 6–8 days to obtain immature BMpDCs. Immature BMcDCs were matured with 1 μg/mL LPS stimulation for 24 h. Immature BMpDCs were matured with 2 μM CpG oligodeoxynucleotide 2216, a ligand of TLR9, for 24 h with or without 1–100 µM nicotine.

### Antigen uptake

Immature BMpDCs were incubated with FITC-conjugated OVA for 24 h with or without nicotine. The effect of nicotine on antigen uptake by BMpDCs was evaluated with flow cytometry analysis.

### Flow cytometry analysis

BMcDCs, BMpDCs, and LPMCs were treated with the following antibody cocktail for 30 min at 4 °C.

- PE-conjugated anti-mouse CD11c antibody
- APC-conjugated anti-mouse mPDCA-1 antibody
- FITC-conjugated anti-mouse CD80 antibody
- PE/Cy7-conjugated anti-mouse CD86 antibody

Flow cytometry analysis was performed using a FACSCanto II (BD Biosciences).

### Apoptosis assay

BMpDCs were incubated with nicotine at a dose of 100 µM for 30 min. The effect of nicotine on BMpDC apoptosis was evaluated with an Annexin V PI apoptosis detection kit according to the manufacturer’s manuals.

### Chemotaxis assay

A BMpDC suspension in 1% FBS/modified RPMI-1640 medium was prepared at a concentration of 1 × 10^6^ cells/mL. One microliter of cell suspension was injected into one side of the chamber of the chemotaxis assay device EZ-TAXIScan™ (GE Healthcare Japan, Tokyo, Japan). A chemokine gradient was generated by injecting 1 µl of CCL21 into the opposite side of the chamber at a concentration of 250 µg/mL. BMpDC migration was recorded every 30 s. The velocity (nm/s), directionality (radians), and number of migrated cells were calculated by a TAXIScan analyzer. Nicotine, Z-VAD-FMK, AZ-10417808 or AG-490 was added to the 1% FBS/modified RPMI-1640 medium at a final concentration of 0.1 to 100 µM in accordance with the study objectives. BMpDCs were incubated for 30 min with MLA at a concentration of 1 µM before the chemotaxis assay.

### Western blot analysis

BMpDCs incubated with or without nicotine for 30 min at 37 °C were suspended in a homogenization buffer composed of 62.5 mM Tris–HCl, 2% SDS, 10% glycerol, 50 mM DTT, and 0.01% bromophenol blue. After sonication, the lysate was incubated for 5 min at 95 °C and then centrifuged at 15,000 rpm and 4 °C for 15 min. The supernatant was subjected to 8% SDS-PAGE at 30 mA. After membrane transfer for 90 min at 125 mA, the membrane was blocked by Block Ace at RT and then washed with TBS-T at RT for 10 min. The membrane was incubated with a rabbit IgG anti-mouse STAT3 antibody or rabbit IgG anti-mouse phospho-STAT3 antibody at RT for 1 h. After being washed with PBS-T at RT for 10 min three times, the membrane was incubated with a solution containing anti-rabbit IgG-HRP and Block Ace at RT for 45 min. Finally, the membrane was washed with PBS-T at RT for 10 min three times, incubated with ECL reagents, and then analyzed using a LAS-4000 (FUJIFILM, Tokyo, Japan).

### Measurement of Caspase-3 activity

BMpDCs were incubated with nicotine at a concentration ranging from 1 to 100 µM for 30 min. Caspase-3 activity was assayed by using the Caspase-3/CPP32 Colorimetric Assay Kit (BioVision) according to the manufacturer’s manuals. The absorbance peak at 450 nm was measured by a plate reader (Life Technologies).

### Measurement of active Rac 1

BMpDCs were incubated with or without 10 µM nicotine or 100 ng/mL CCL21 for 30 min at 37 °C. After incubation, the activation of Rac 1 in BMpDCs was evaluated by the Active Rac 1 Pull-Down and Detection Kit according to the manufacturer’s manuals.Total Rac 1 and active Rac 1 levels were calculated with ImageJ software (Camera: LAS-4000, FUJIFILM).

### Statistical analyses

Data are shown as the mean ± SEM. The statistical significance of differences between 2 independent groups was evaluated by 2-tailed unpaired Student’s *t*-test. For 3 or more independent groups, one-way ANOVA, followed by Dunnett’s multiple comparison test was used to evaluate the statistical significance of differences. A *P* value (*P*) < 0.05 was defined as statistically significant.

## Supplementary Information

Supplementary Legends.

Supplementary Figures.
