# Supplementary material for: Cholinergic anti-inflammatory pathway ameliorates murine experimental Th2-type colitis by suppressing the migration of plasmacytoid dendritic cells
Source: Sci Rep. 2022 Jan 7;12:54. doi: 10.1038/s41598-021-04154-2 (PMC8742068; doi:10.1038/s41598-021-04154-2)
Supplement: Supplementary file 1 — Supplementary Legends. [file 41598_2021_4154_MOESM1_ESM.docx]

**Supplementary Figure Legends**

Supplementary Figure 1

OXZ colitis was established and evaluated. (**A**) OXZ mice exhibited weight loss (the typical mean weight change during the development of colitis is shown). (**B** and **C**) The mRNA expression of Th1 and Th2 cytokines in the spleen (**B**) and colon (**C**) was measured by real-time PCR. The mRNA expression of IFN-γ was significantly decreased, and that of IL-4, IL-5, and IL-10 was significantly increased in the spleen of OXZ mice compared with that of control mice (**B**). The mRNA expression of IFN-γ, IL-4, IL-5, and IL-10 mRNA was increased in the colon of OXZ mice compared with that of control mice (**C**). Data are represented as the mean value ± SEM. *n* = 4–9. **P* < 0.05, ***P* <0 .01 vs normal mice. *P* values were calculated using 2-tailed unpaired Student’s *t*-test.

Supplementary Figure 2

OXZ colitis developed and its severity was assessed. (**A** and **B**) Difference in the development of colitis between BALB/c and C57BL/6 mice were evaluated. BALB/c mice showed weight loss, while C57BL/6 mice did not (**A**; the typical mean weight change during the development of colitis is shown). The DAS and CDS of C57BL/6 mice were significantly lower than those of BALB/c mice (**B**; *n* = 8–21, ***P* < 0.01 vs BALB/c mice). (**C** and **D**) The effect of FK506 administration on the development of colitis was evaluated. Treatment with FK506 aggravated weight loss (**C**; the typical mean weight change during the development of colitis is shown), the DAS, and the CDS in OXZ mice (**D**; *n* = 10 for each group, ***P* < 0.01 vs OXZ mice). (**E**) The effect of 5-ASA administration on the development of colitis was evaluated. Treatment with 5-ASA did not improve the DAS or CDS (*n* = 10 for each group). (**F**) The effect of prednisolone administration on the development of colitis was evaluated. The DAS, the CDS, and MPO activity in OXZ mice treated with prednisolone were significantly decreased compared with those in OXZ mice (*n* = 8 for each group, **P* < 0.05, ***P* < 0.01 vs OXZ mice). Data are represented as the mean value ± SEM. **P* < 0.05, ***P* <0 .01 vs normal mice. *P* values were calculated using 2-tailed unpaired Student’s *t*-test.

Supplementary Figure 3

The mRNA expression of α7nAChRs in DCs was measured by RT-PCR. The full-length gel images with three different exposure times are shown. mPDCA-1^+^CD11C^int^ pDCs collected from the lamina propria of the colon in OXZ mice, and immature and mature mouse BMpDCs expressed α7nAChR mRNA.

Supplementary Figure 4

The effects of nicotine on the maturation and uptake capacity of BMpDCs were examined by flow cytometry. (**A** and **C**) The effect of nicotine on the uptake capability was measured by adding FITC-conjugated OVA to BMpDC culture medium. The number of FITC^+^ BMpDCs exhibited little difference with or without nicotine (**A**: a representative experiment is shown, **C**: *n* = 3 for each group). (**B** and **D**) The frequency of CD80^+^CD86^+^ cells in BMpDCs was significantly increased by treatment with CpG oligodeoxynucleotides. The increased expression of CD80 and CD86 was not inhibited by nicotine at doses of 1–100 µM (**B**: a representative result is shown, **D**: *n* = 3 for each group). Data are represented as the mean value ± SEM. *P* values were calculated using one-way ANOVA with Dunnett’s multiple comparison test (**C** and **D**).

Supplementary Figure 5

The expression of active Rac 1 (Rac1-GTP) and total Rac 1 in BMpDCs was measured by western blotting. The representative full-length blots with three different exposure times (3 sec, 60 sec and 120 sec) are shown. The membrane was incubated with ECL reagents, and then analyzed using a LAS-4000. To clearly indicate the images used in Figure 4, red boxes around these parts were put in the supplementary Figure 5. The western blot images with the shortest exposure time (3 sec) were used for quantitative data analysis.

Supplementary Figure 6

The ratio of phosphorylated STAT3 to total STAT3 in BMpDCs was significantly increased by stimulation with nicotine, as determined by immunoblotting. The representative full-length blots with three different exposure times (10 sec, 30 sec and 10 min) are shown. The membrane was incubated with ECL reagents, and then analyzed using a LAS-4000. To clearly indicate the images used in Figure 6, red boxes around these parts were put in the supplementary Figure 6. The western blot images with the shortest exposure time (10 sec) were used for quantitative data analysis.

Supplementary Figure 7

The effects of nicotine on BMpDC death were examined by flow cytometry. To discriminate between apoptotic and necrotic cell death, BMpDCs were stained with Annexin V and PI. The frequencies of Annexin V^+^PI^-^ BMpDCs and Annexin V^+^PI^+^ BMpDCs were not affected by nicotine at a dose of 100 μM (a representative experiment is shown, *n* = 5 for each group).
