# Supplementary material for: Cholinergic anti-inflammatory pathway ameliorates murine experimental Th2-type colitis by suppressing the migration of plasmacytoid dendritic cells
Source: Sci Rep. 2022 Jan 7;12:54. doi: 10.1038/s41598-021-04154-2 (PMC8742068; doi:10.1038/s41598-021-04154-2)
Supplement: Supplementary file 2 — Supplementary Figures. [file 41598_2021_4154_MOESM2_ESM.pptx]

## Slide 1
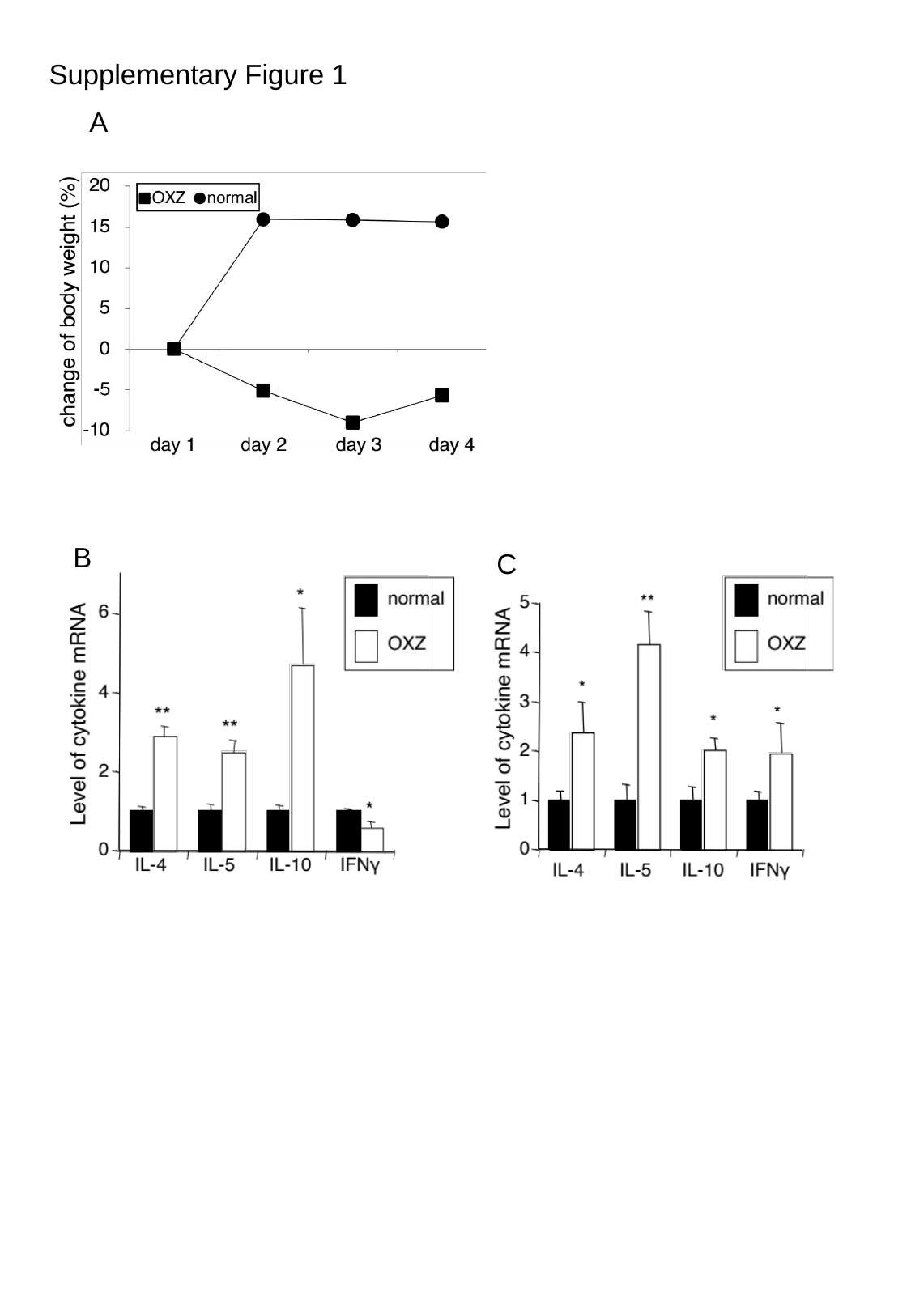

Supplementary Figure 1
A
B
C

## Slide 2
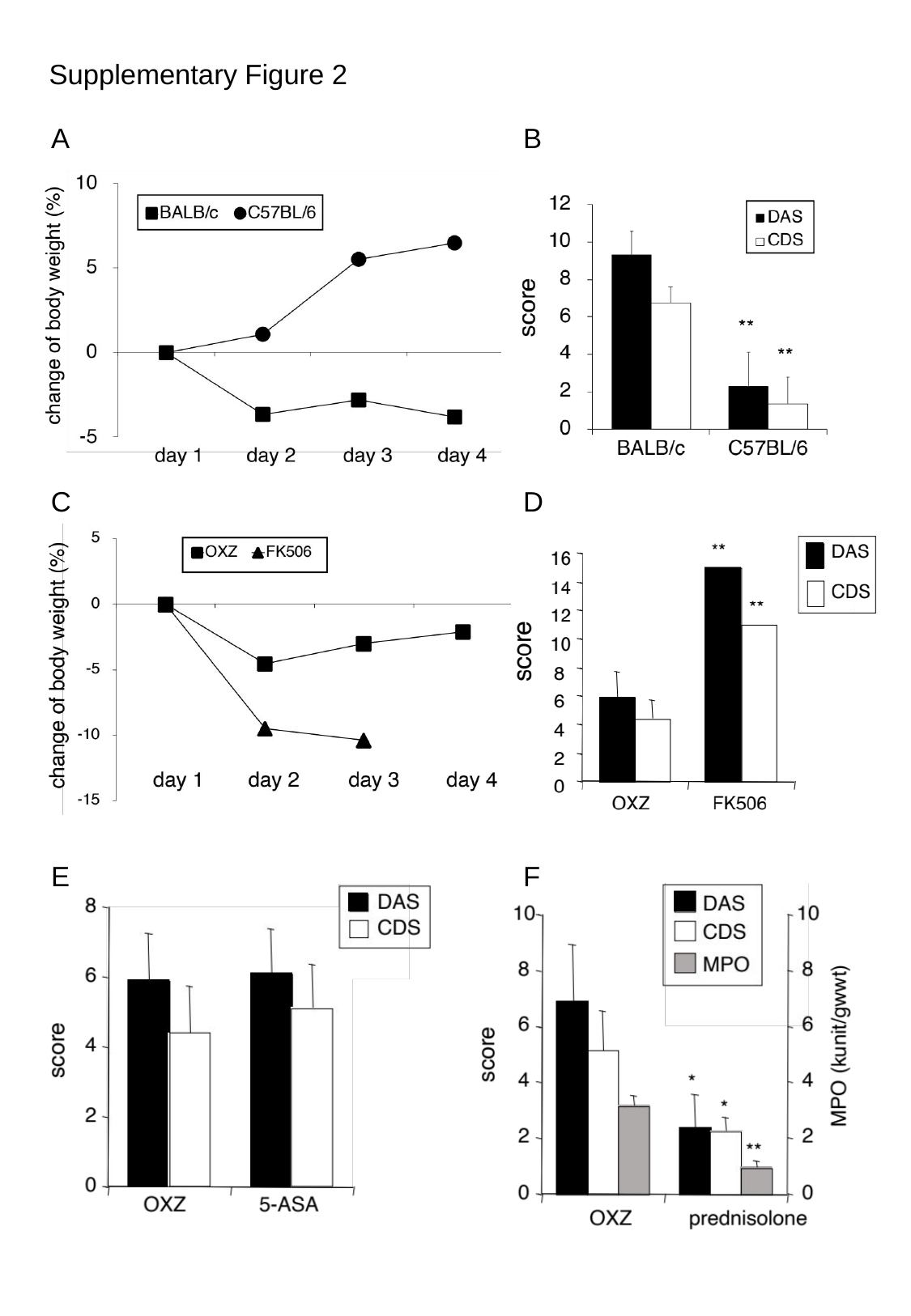

Supplementary Figure 2
A
B
C
D
E
F

## Slide 3
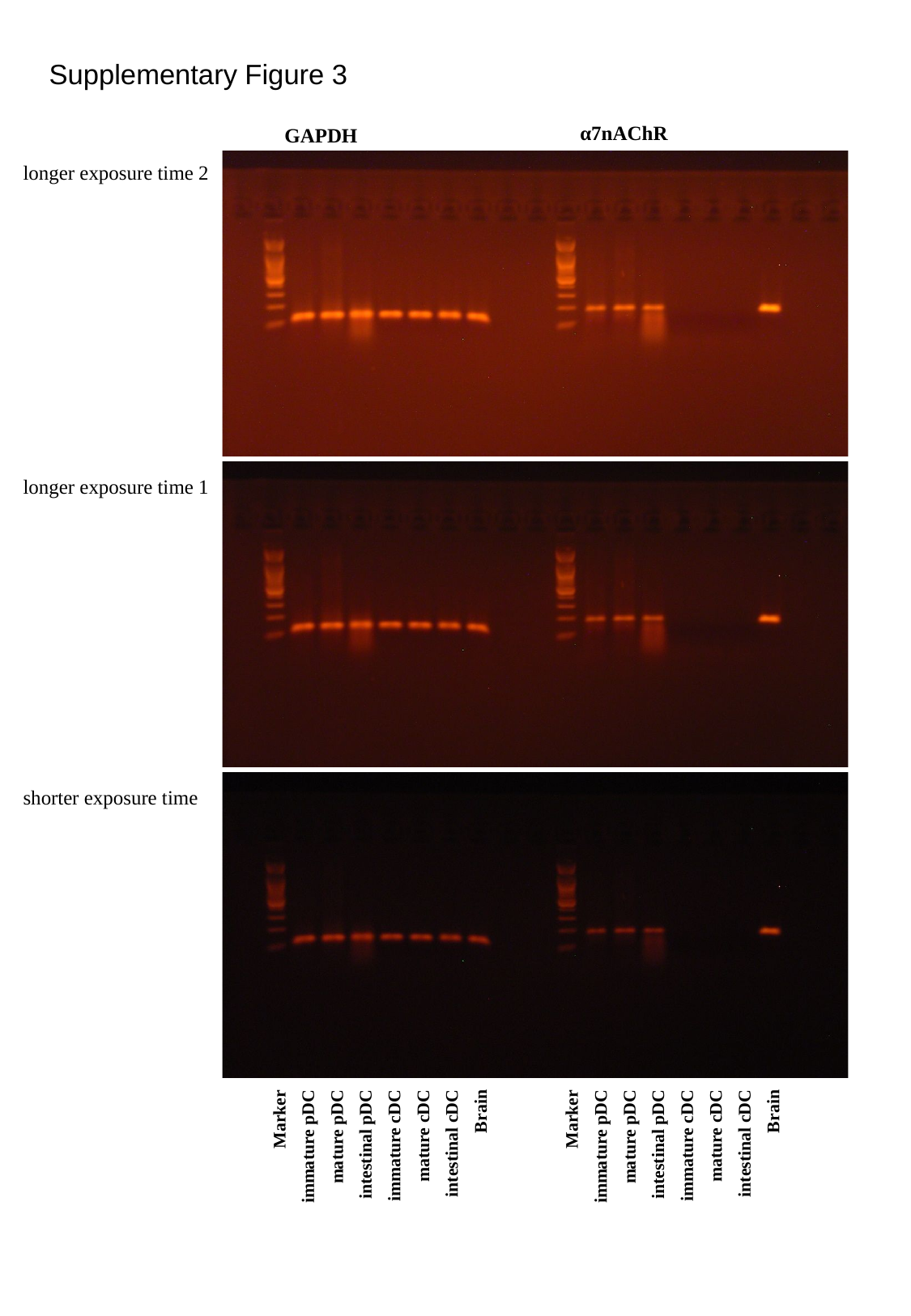

Supplementary Figure 3
α7nAChR
GAPDH
longer exposure time 2
longer exposure time 1
shorter exposure time
Brain
Brain
mature cDC
mature cDC
mature pDC
mature pDC
intestinal cDC
intestinal cDC
intestinal pDC
intestinal pDC
immature cDC
immature cDC
Marker
immature pDC
Marker
immature pDC

## Slide 4
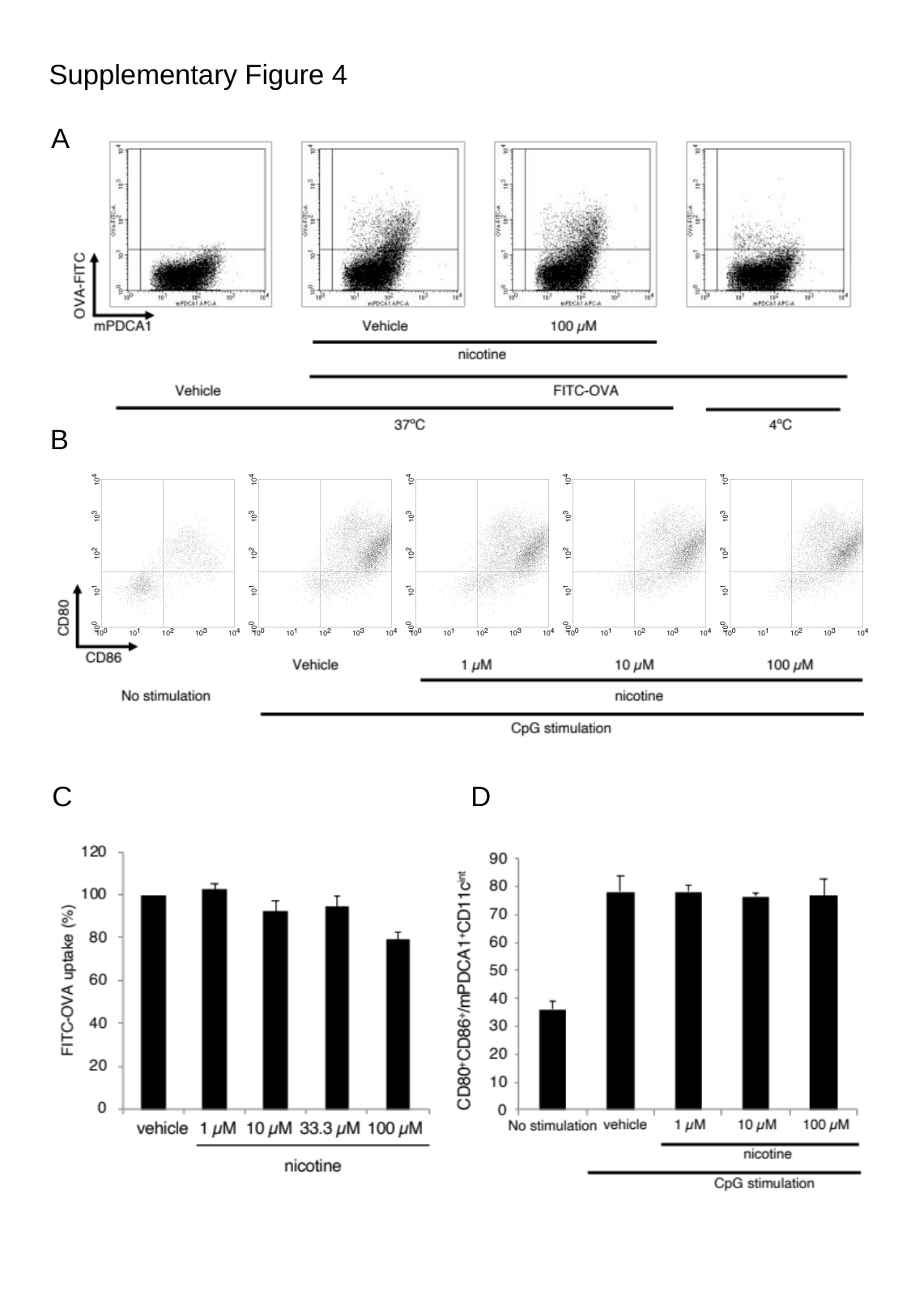

Supplementary Figure 4
A
B
C
D

## Slide 5
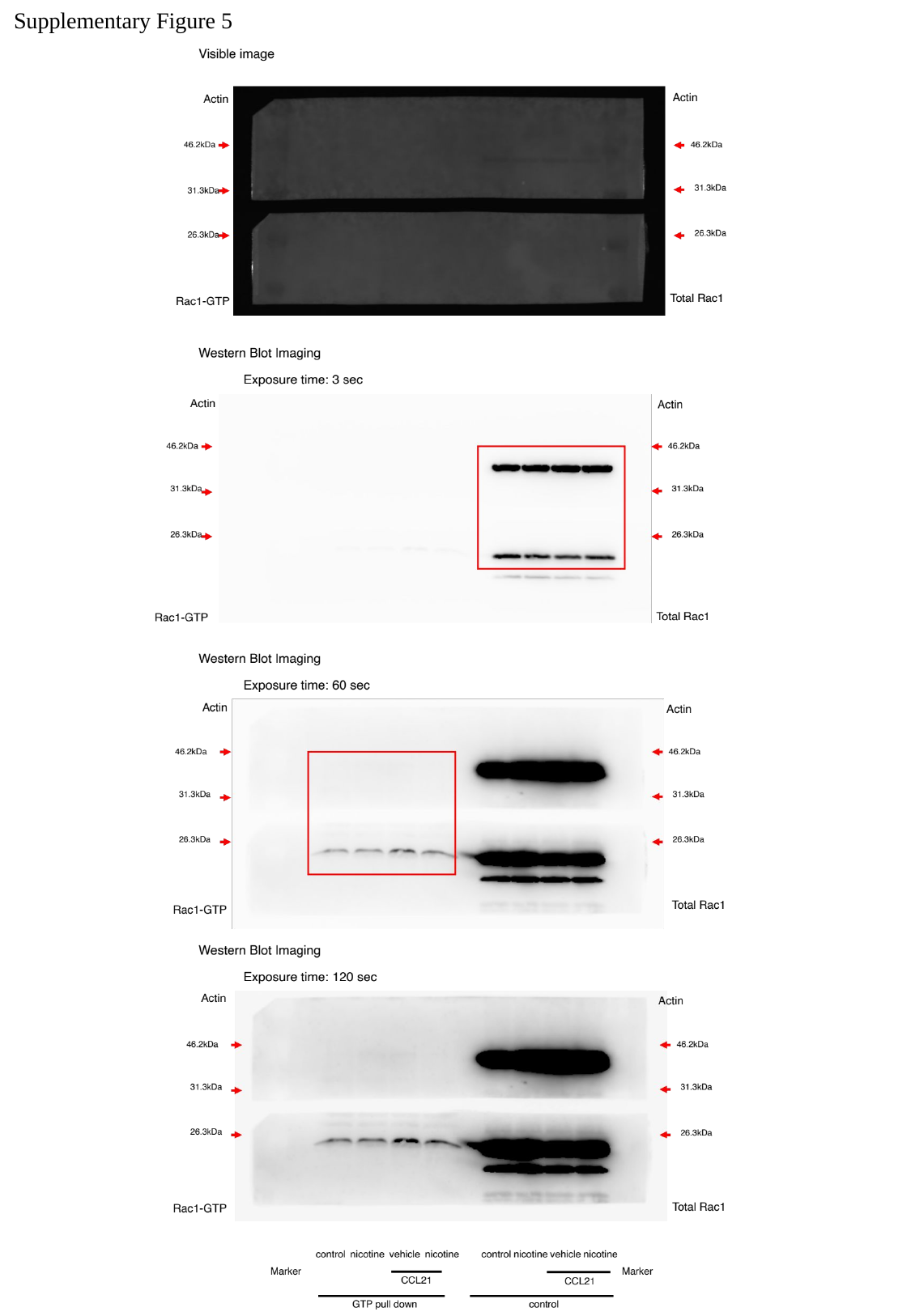

Supplementary Figure 5

## Slide 6
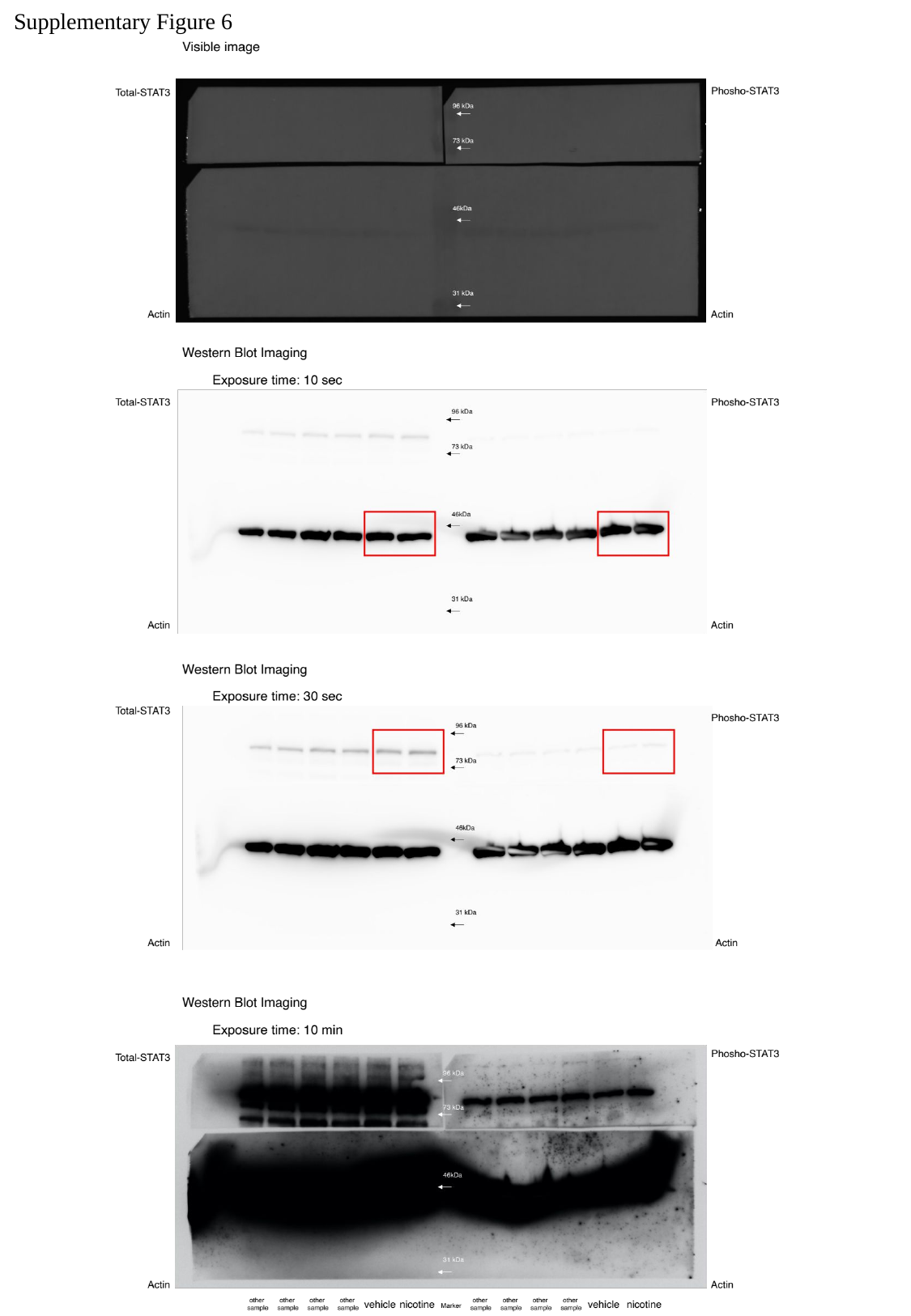

Supplementary Figure 6

## Slide 7
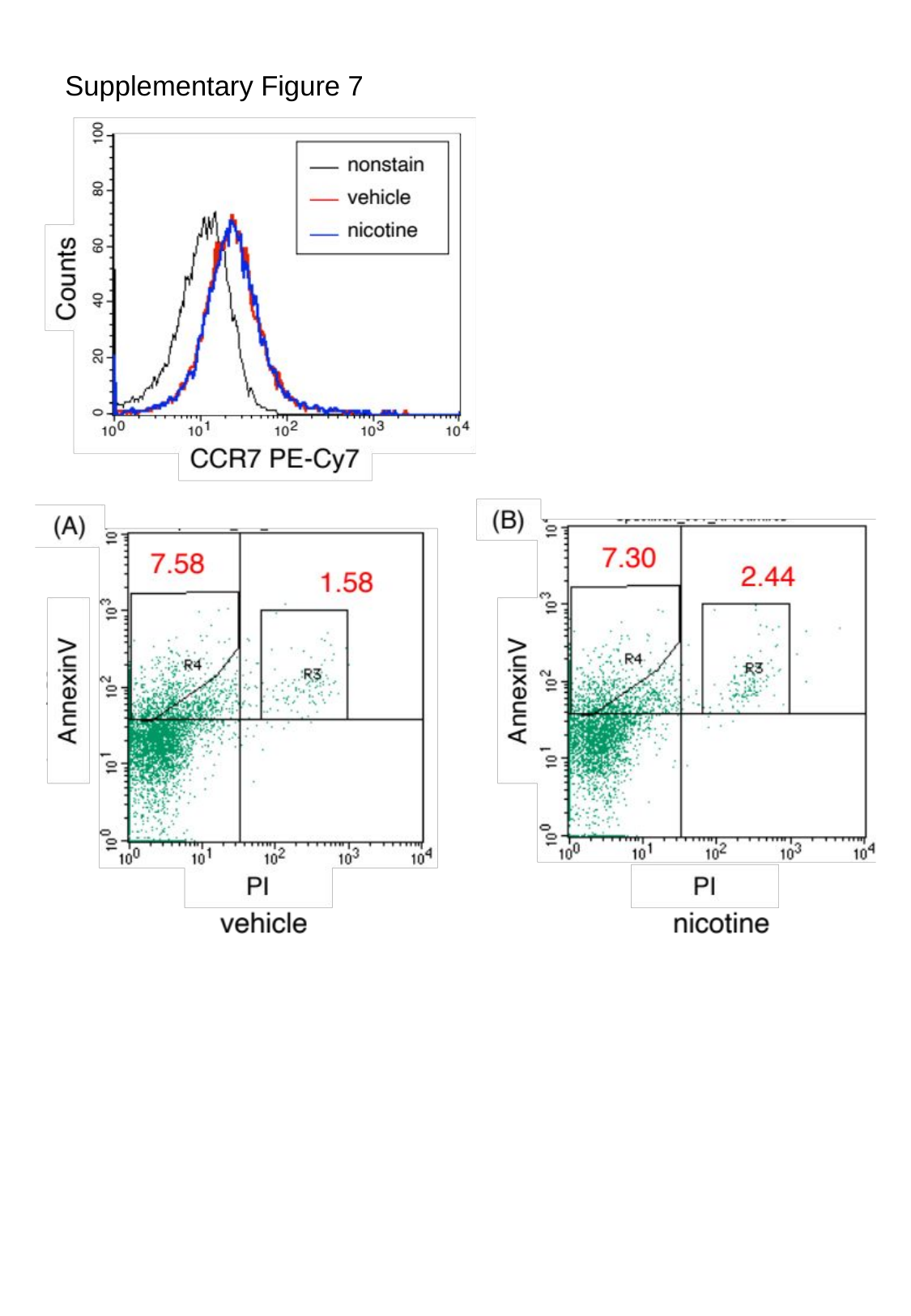

Supplementary Figure 7
